# Novel Method of Synthesis of 5''-Phosphate 2'-*O*-ribosyl-ribonucleosides and Their 3'-Phosphoramidites

**DOI:** 10.3390/molecules181214780

**Published:** 2013-11-29

**Authors:** Marcin K. Chmielewski, Wojciech T. Markiewicz

**Affiliations:** Institute of Bioorganic Chemistry, Polish Academy of Sciences, Noskowskiego 12/14, Poznan PL-61704, Poland; E-Mail: maro@ibch.poznan.pl

**Keywords:** 2'-*O*-ribosylribonucleosides, ribosylation, methionine tRNA, initiator tRNA, *H-*phosphonate, phosphoramidite, nucleosides, nucleotides

## Abstract

Synthesis of 5''-phosphate 2'-*O*-ribosylribonucleosides [*Nr(p)*] of four common ribonucleosides, and 3'-phosphoramidites of 5''-phosphate 2'-*O*-ribosyladenosine and 2'-*O*-ribosylguanosine using the *H-*phosphonate chemistry is described. An additional ring protected by benzoyl groups was incorporated into the main ribosyl ring in the reaction with 1-*O*-acetyl-2,3,5-tri-*O*-benzoyl-*β*-d-ribofuranose in the presence of SnCl_4_. The obtained 2'-*O*-ribosylribonucleosides (*Nr*) were applied in the subsequent transformations with selective deprotection. Ethanolamine was applied as a very convenient reagent for selective removal of benzoyl groups. Additionally, the tetraisopropyldisiloxane-1,3-diyl (TIPDSi) group was found to be stable under these deprotection conditions. Thus, the selectively deprotected 5''-hydroxyl group of *Nr* was transformed into an *H-*phosphonate monoester which was found to be stable under the following conditions: the removal of the TIPDSi group with triethylammonium fluoride and the dimethoxytritylation of the 5''-hydroxyl function. The 5''-*H-*phosphonate of *Nr* precursors was easily transformed to the corresponding dicyanoethyl 5''-*O*-phosphotriesters before phosphitylation, which gave 3'-phosphoramidite units of *Nr(p)* in high yield. The derived phosphoramidite units were used in an automated oligonucleotide synthesizer to produce dimer *Ar(p)T* via the phosphoramidite approach. The obtained products were fully deprotected under standard deprotection conditions giving dimers with a 5''-phosphate monoester function. Application of an alkaline phosphatase to prove the presence of an additional phosphate group was described.

## 1. Introduction

The 2'-*O*-ribosylribonucleosides, which are the most common natural representatives of disaccharide nucleosides, were found as minor nucleosides in tRNA with a key function in protein biosynthesis. In 1991, the first of the new 2'-*O*-ribosylribonucleosides from initiator tRNA^Met^ was isolated and identified as *β*-anomer of 5''-phosphate of *O*-ribosyl(1''→2'') adenosine (*Ar(p)*) [1]. Another minor nucleotide: 5''-phosphate of *O*-ribosyl(1''→2'') guanosine (*Gr(p)*) [2] was identified soon afterwards. Both of these modified nucleosides are located in the T-stem of the yeast tRNA_i_^Met^ at position 64 of their sequence. It has been shown that this modification plays an important role in the initiation of protein biosynthesis [3]. The primary feature of this process is the interaction of tRNA with protein factors eEF-1α and elF-2. The presence of 2'-*O*-ribosyl modified nucleoside in tRNA determines the functions of methionine tRNA as initiation tRNA [4]. Discovery of this important modification in natural products started the investigation process aimed at their possible applications. In the first place it initiated their chemical synthesis [5] and a method for investigating the enzymes of nucleic acid metabolism [6,7].

Markiewicz and co-workers were the first to develop an effective chemical synthesis pathway for 2'-*O*-ribosylribonucleosides using ribosylation of 3',5'-protected nucleosides [5]. Studies of the chemical synthesis of non-phosphorylated 2'-*O*-ribosyl nucleotides were extended by the synthesis of its dimer containing 2'-*O*-ribosyl nucleotide. The structural studies of these 2'-*O*-ribosyl ribonucleosides using X-ray and NMR analysis as well as the synthesis of 3'-phosphoramidites of fully protected 2'-*O*-ribosyloribonucleosides and the solved crystal structure for 2'-*O*-ribosyluridine were also described [8]. The same method for synthesis of non-phosphorylated 2'-*O*-ribosyl nucleosides was then used by Mikhailov *et al.* [9,10] and Carell *et al.* [11] in their pathway to disaccharide nucleosides. The 2'-*O*-*β*-d-ribopyranosylcytidine is another example of disaccharide nucleosides for which the abovementioned authors have reported the crystal structure [12].

Although the first synthesis of 2'-*O*-*β*-d-ribofuranosyl-(1''-2')-adenosine-5''-*O*-phosphate [13,14] and 2'-*O*-*β*-d-ribofuranosyl-(1''-2')-guanosine-5''-*O*-phosphate [15] was developed, the yields were low. These compounds were also applied in chemical synthesis of nucleic acids and incorporated in RNA sequences with other minor components of tRNA [16]. This modification has no effect on thermal stability of the duplex, and the extra ribose moiety seems to be stabilized by bridged *H-*bonds [17]. Enzymatic incorporation of 2'-*O*-ribosylribonucleoside residue into oligonucleotides was also investigated [18].

## 2. Results and Discussion

### 2.1. Synthesis of 5″-Phosphate 2'-O-ribosylribonucleosides

Herein we present for the first time a different efficient pathway of the synthesis of 5''-phosphorylated 2'-*O*-ribosylribonucleotides for all four nucleosides, and the corresponding 3'-phosphoramidites of adenosine and guanosine. Chemical synthesis of 5''-phosphorylated disaccharide nucleosides requires choosing a proper protecting system in order to handle the phosphate functional groups that are to be introduced into different positions and handled differently throughout the synthesis of monomers and their later introduction into oligonucleotides. Taking this into account, we applied [5] the bifunctional TIPDSi protecting group for the protection of the 3'- and 5'-positions of the ribonucleoside substrate, which resulted in an effective ribosylation of the 2'-hydroxyl function with 1-*O*-acetyl-2,3,5-tri-*O*-benzoyl-*β*-d-ribofuranose as the first step of our synthetic scheme. The known methods for removal of ester groups could hardly guarantee high yield. One would rather expect little selectivity or removal of all protecting groups that are in fact base labile protections. In order to perform further transformations we chose a pathway that would allow us to remove all ester groups from the additional ribose ring, including the nucleobase exo-amine protecting group, while the TIPDSi group remained stable.

However, we found that treatment of fully protected ribosylribonucleosides **1** with concentrated ammonium hydroxide leads to the removal of benzoyl (and isobutyryl from **1c**) groups and partial cleavage and removal of the TIPDSi group. Using *n*-butylamine under mild conditions was also not useful. In light of the above-mentioned non-selectivity ethanolamine was applied as a weaker base. Thus, benzoyl groups from fully protected nucleosides **1a**–**d** could be selectively removed with an alcoholic solution of ethanolamine and the following order of removal was observed: NHBz > 5″-OBz > 2''-,3''-OBz. The process is slow enough for individual deprotection stages to be easily followed by thin layer chromatography (TLC). Under the applied conditions the disiloxane TIPDSi group was stable, which was additionally confirmed by the full NMR analysis. Despite some selectivity observed in the removal of ester groups we decided to remove these protections completely. This method allows one to functionalize an additional sugar ring while the hydroxyl function in the main ring remains protected, which enables selective introduction of phosphate in the 5'' position.

**Scheme 1:**
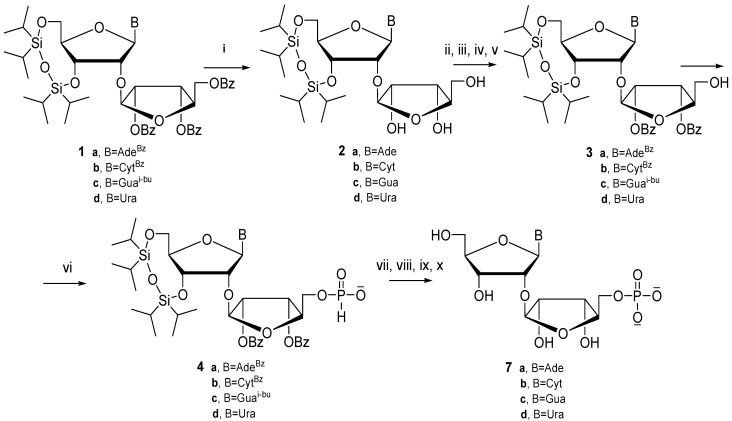
Synthesis of 5''-phosphate 2'-*O*-ribosylribonucleosides (**5**).

The applied solvent also had a significant effect on speed and selectivity of benzoyl group removal. When ethanolamine was used in ethanol as a solvent, benzoyl groups were removed very fast, while increasing the length of the aliphatic chain of the alcohol slowed the reaction down considerably. In this context the best result was obtained for isopropyl alcohol. In the next step it was necessary to reintroduce the N-acyl protecting groups. The standard protection method with transient trimethylsilyl protection turned out to be fully applicable. Thus, the TIPDSi-protected *N*-acyl-2'-*O*-ribosylnucleosides were obtained in high yields (72%–89%). These derivatives were then protected at 5''-position using 4,4'-dimethoxytrityl chloride in pyridine. The tritylation step was followed by benzoylation of the 2''- and 3''-hydroxyl functions. Finally, acid treatment with dichloroacetic acid to remove the DMT group from 5″-position gave the TIPDSi-protected *N*-acyl-2'-*O*-(2'',3''-di-*O*-benzoyl)ribosylribonucleosides **3** ready to incorporate the phosphate function (Scheme 1). In the case of synthesis of the uridine derivative the transient TMS protection and base acylation reaction were not necessary and **2d** was first 5''-*O*-dimethoxytritylated, then 2''- and 3''-O-benzoylated, and finally detritylated to obtain the target compound **3d**. The overall reaction yields were quite high and this shows clearly the advantage of our reaction scheme to obtain **3** in comparison with the Mikhailov *et al.* approach based on the use of 1,2,3-tri-*O*-benzoyl-5-*O*-(phenoxyacetyl)-*β*-ribofuranose [19]. Our approach is based on reagents that are all easily and commercially available.

*Reagents*: (i) 2-aminoethanol, i-PrOH; (ii) TMSCl, pyridine; (iii) BzCl, pyridine; (iv) DMTCl, pyridine; (v) DCA, dichloromethane; (vi) (Ph)_2_PHO; (vii) 3-hydroxypropionitrile, PivCl, pyridine; (viii) H_2_O/I_2_,pyridine; (ix) TEAHF/THF; (x) NH_4_OH/pyridine.

The next step to introduce the phosphate function into the 5''-position was achieved by phosphitylation of compounds **3** with diphenyl phosphite according to Kraszewski *et al.* [20,21] We chose the *H-*phosphonate approach for this functionalization as it should provide easy access to both 5''-phosphate monoester of 2'-*O*-ribosylribonucleosides as well as their 5''-phosphotriester. The latter option looked very promising and should allow us to obtain 3'-phoshoramidites of fully protected 2'-*O*-ribosylribonucleosides with the 5″-phosphotriester function included. This would solve the problem of introduction of natural, non-dephosphorylated 2'-*O*-ribosylribonucleoside units into oligo-nucleotide chains.

The *H-*phosphonate function was introduced into the 5''-position of **3** and the corresponding 5''-(*H-*phosphonates) **4** were obtained in high yields. The subsequent further transformation to phosphate should not be difficult as it was reported that trimethylsilyl *H-*phosphonate diesters can be oxidized under mild conditions [22]. Thus, the 5''-*H-*phosphonate monoesters **4** could be oxidized to corresponding 5''-phosphates with two following procedures: in the first approach iodine in aqueous pyridine was used after their prior reaction with trimethylsilyl chloride in anhydrous pyridine, and the second approach took advantage of the reaction of 5''-(*H-*phosphonates) **4** with 3-hydroxypropionitrile after activation with pivaloyl chloride in pyridine (Scheme 1). The latter approach is a typical coupling reaction used in the synthesis of oligonucleotides via the *H-*phosphonate approach. The obtained 2-cyanoethyl *H-*phosphonate diester was again oxidized with iodine in aqueous pyridine to give 5''-(2-cyanoethyl)phosphate diesters. In both cases the final deprotection using fluoride (triethylammonium fluoride) to remove the TIPDSi group and treatment with concentrated ammonia at higher temperature provided natural 5''-phosphates *O*-ribosyl(1''→2'') of four native ribonucleosides **7** in high yields [23]. The structures of the final 5″-phosphates **7** were corroborated with ^1^H-, ^13^C- and ^31^P-NMR and mass spectroscopy. Thus, the new way of synthesis of 5''-phosphates of 2'-*O*-ribosylnucleosides was developed.

### 2.2. Synthesis of 3'-O-Phosphoramidites of 5″-Phosphate 2'-O-ribosylribonucleosides and Their Use in the Preparation of a Dinucleoside Monophosphate

**Scheme 2:**
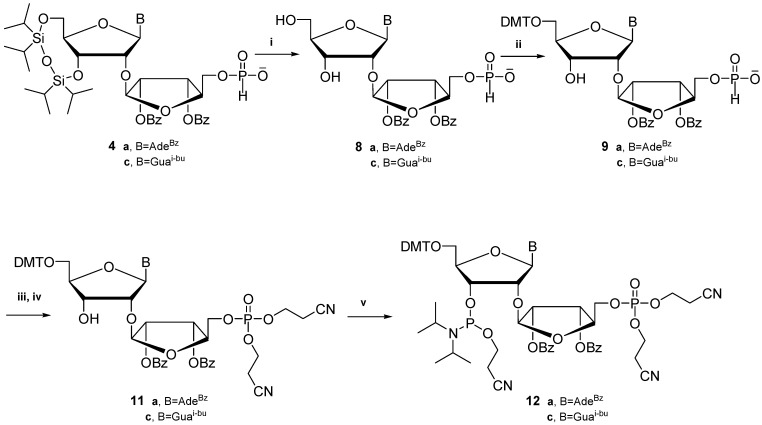
Synthesis of 3'-phosphoramidite of 5''-phosphate 2'-*O*-ribosylribonucleosides (**12**).

Although the new procedure developed by us allows one to obtain unprotected 5″-phosphates of 2'-*O*-ribosylribonucleotides (**7**), there is no procedure developed to incorporate these modifications into nucleic acid sequences. Therefore, we decided to develop a method that would make such a synthesis possible and use a phosphoramidite approach. The presence of the *H-*phosphonate monoester is known to interfere with both the coupling reaction and the phosphoramidite synthesis. However, the *H-*phosphonate monoester is not stable during the phosphitylation reaction. Therefore, before final incorporation of 3'-phosphoramidite, it was necessary to convert the 5''-*H-*phosphonate monoesters into the corresponding 5''-phosphotriesters. This goal was achieved only for purine nucleosides (adenosine and guanosine), the same for which the 2'-*O*-ribosyl modifications was found in tRNA (Scheme 2). In the next chemical pathway the obtained 5''-*H-*phosphonate monoesters **4** of nucleotides were used. Further steps needed appropriate arrangement of the protecting groups wherefore protected 2'-*O*-ribosylribonucleosides were first treated with fluoride reagent to remove the TIPDSi group. Then, the resulted 5''-*H-*phosphonates of N-protected 2'',3''-di-*O*-benzoyl of 2'-*O*-ribosyladenosine and guanosine (compounds **8a** and **8c**) reacted with dimethoxytrityl chloride in pyridine. The desired 5'-*O*-dimethoxytrityl derivatives **9a** and **9c** were obtained. This shows that *H-*phosphonate as the precursor of the phosphate function proved to be compatible with tritylation conditions. The obtained derivatives were converted in the one-pot procedure into desired 5''-(bis-2-cyanoethyl)phosphotriesters **11a** and **11c** by firstly coupling with 3-hydroxypropionitrile activated by pivaloyl chloride in pyridine and next the intermediates cyanoethyl 5''-(*H-*phosphonate)diesters of adenosine (**10a**) and guanosine (**10c**) were converted to **11a** and **11c**, respectively, followed by oxidative coupling with iodine [24]. The progress of the reaction was followed by ^31^P-NMR analysis. The coupling proved to be quite fast and effective. The latter reaction went to completion in 10 min. at room temperature, which was also shown by ^31^P-NMR analysis. Finally, the compounds **11a** and **11c** were submitted to the 3'-phosphitylation procedure with bis(diisopropylamino)(2-cyanoethoxy)phosphine and 1-*H*-tetrazole as activator and desired 3'-phosphoramidites **12** were obtained in satisfying yields.

*Reagents*: (i) TEAF/THF; (ii) DMTCl, pyridine; (iii) 3-hydroxypropionitrile, PivCl, pyridine; (iv) 3-hydroxypropionitrile/I_2_ in pyridine; (v) bis(diisopropylamino)(2-cyanoethoxy)phosphine, 1-*H*-tetrazole.

**Figure 1:**
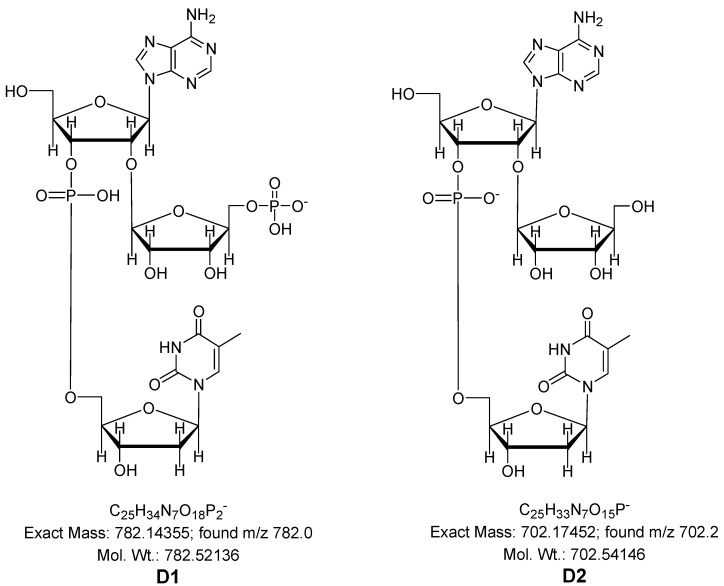
Structures of dimers with mass calculations.

The usefulness of 3'-phosphoramidite for 2'-O-ribosylnucleosides has been demonstrated through the use of the obtained 3'-phosphoramidite of 5''-phosphate 2'-*O*-ribosyladenosine (**10**) in the synthesis of dimer *Ar(p)T* (**D1**, Figure 1). The coupling time was extended as one might expect on the basis of our previous studies [25] showing that a bulky 2'-*O*-substituent can pose a serious steric hindrance in the coupling reaction. The synthesis was performed using the solid support approach. The standard synthesis protocol was applied, except for the coupling step which was extended to 12 min.

The synthesis of dimer *ArT* (**D2**) with 2'-*O*-ribosyl ribonucleosides without the 5″-phosphate function was performed using phosphoramidite which was synthesized by the previously described procedure [8]. Both synthesized dimers were removed from the support and deprotected using the standard procedures used in the chemical synthesis of oligonucleotides by the phosphoramidite approach. The crude products were analyzed and purified by means of HPLC.

**Figure 2:**
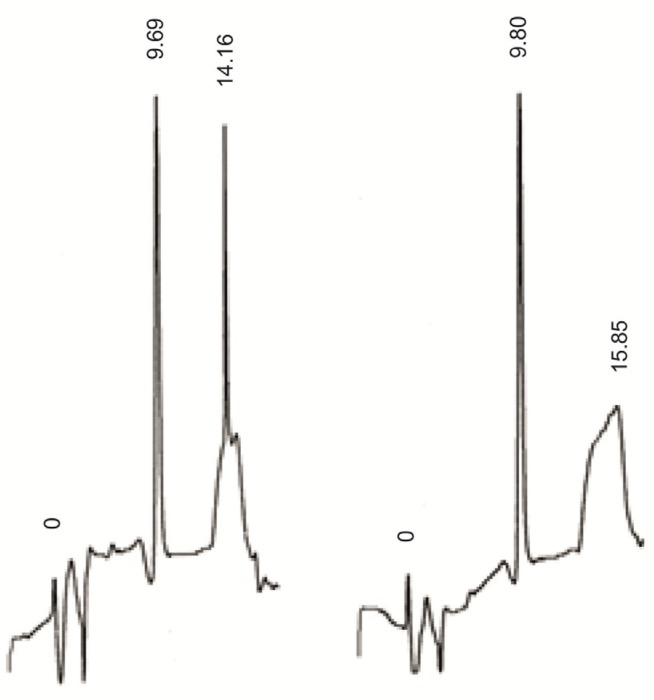
HPLC analysis of enzymatic digestion of the dimer **D1** and compared with the dimer **D2**.

In order to confirm the presence of the 5''-phosphate attached to the 2'-*O*-ribosylnucleoside unit the dimer **D1** was treated with alkaline phosphatase and the process was followed by the HPLC (Figure 2) that showed disappearance of **D1** with the retention time of 14.1 min. and the formation of a product with retention time identical as that of **D2** (9.8 min). Total removal of phosphate from **D1** resulted in a dimer without the 5''-phosphate that is identical with the dimer **D2**. The massive amounts of impurities shown in the chromatographs result from the protein buffers applied, not from the synthesized dimers. The analysis confirming dimer purity has been included in the supporting information. It finally proved that the developed and reported here procedure allows for the first time introduction of natural 2'-*O*-ribosylnucleoside-5''-phosphates into dinucleoside monophosphate structure.

The analysis of the mixture of both dimers(**D1** and **D2**) before (left) and after alkaline phosphatase digestion (right). Numbers at peaks denote retention time t_R _[min] (**D1**—14.16 min, **D2**—9.8 min), detection at 256 nm; analysis conditions: starting from 0.1 M TEAA pH = 7.0, a linear gradient of 0.5% MeCN/min, flow rate 1 mL/min. time 40 min, C-18 Hypersil column, temp. 25 °C.

## 3. Experimental

### 3.1. General Methods

All reagents were commercially available and the synthesis of **1** has been described previously [5]. Solvents except for methanol were used without further purification. Triethylammonium fluoride (TBAHF) in anhydrous THF was prepared as 1 M solution according to [26]. Reaction progresses were usually monitored with TLC using Merck silica gel 60 F254 plates and UV light at 254 nm. All chromatographic purifications were carried out on the silica gel 60 0.0063 e 0.200 mm (Merck) and on the silica gel 60 0.040 e 0.063 mm or on the silanized silica gel 60 0.063 and 0.200 mm (Merck). For ^1^H-NMR (300 MHz) and ^13^C-NMR (75 MHz) tetramethylsilane (TMS) was used as an internal standard. ^31^P-NMR spectra were recorded at on Varian Unity 300 spectrometer operating at 121 MHz; 5% H_3_PO_4_ in D_2_O reference was used as an external. Fast atom bombardment mass spectrometry (FAB-MS) spectra were obtained on two sector mass spectrometers of reverse B/E geometry. A CsI gun supplied the primary ion beam (12 keV, Cs+). The secondary ion beam was accelerated to 8 kV. The compound was dissolved in 3-nitrobenzyl alcohol.

### 3.2. General Procedure for the Synthesis of 3',5'-O-(Tetraisopropyldisiloxane-1,3-diyl)-2'-O-β-d-ribo-furanosyl Ribonucleosides **2**

Ethanolamine (10 mL) was added to a solution of the protected 2'-*O*-ribosylribonucleoside **1** (475 mg) in isopropyl alcohol (45 mL). The reaction mixture was kept for 72 h in ambient temperature. TLC analysis monitored the progress of the reaction during this time. After debenzoylation was completed the solution was concentrated and the reaction mixture was partitioned between water (15 mL) and dichloromethane (25 mL). Methanol (1 mL) was added to the organic layers, dried and concentrated under reduced pressure, and the residue was put on top of a silica gel column and purified by chromatography in a gradient of methanol (1%–5%) in dichloromethane to give a pure product with the following yields: **2a**: yield 82%; FAB-MS: *m/z* [M−H]^+^; Calcd for C_27_H_48_N_5_O_9_Si_2_^+^, 642.30; found *m/z* 642.2; ^1^H-NMR (300 MHz, CDCl_3_): δ (ppm) 8.15 (s, 1, H-2); 8.07 (s, 1, H-8); 7.40 (s, 2, NH_2_); 5.99 (s, 1, H-1'); 5.07 (s, 1, H-1″); 4.97 (m, 1, H-3'); 4.62 (d, 1, H-2'); 4.11 (m, 1, H-3''); 4.08 (d, 1, H-5'); 3.95 (m, 1, H-4'); 3.92 (d, 1, H-5'); 3.85 (d, 1, H-2''); 3.75 (m, 1, H-4″); 3.59 (2×d, 1, H-5''); 3.39 (2×d, 1, H-5''); 1.11–1.05 (m, 28, TIPDSi); ^29^Si-NMR (CDCl_3_): δ (ppm) −9.59, −10.78.

**2b**: yield 89%; ^1^H-NMR (CDCl_3_): δ (ppm) 7.92 (s, 1, H-8); 5.79 (s, 1, H-1'); 5.37 (s, 1, H-1''); 4.49 (m, 2, H-3', 3''); 4.37 (d, 1, H-2'); 4.25 (d, 1, H-2''); 4.16–4.10 (m, 2, H-4', 5); 4.04–3.92 (m, 3,H-4'', 5'', 5'); 3.73 (2×d, 1, H-5''); 1.11–0.98 (m, 28, TIPDSi).
**2c**: yield 72%; ^1^H-NMR (CDCl_3_): δ (ppm) 7.84 (d, 1, H-6); 5.81 (d, 1, H-5); 5.54 (s, 1, H-1'); 5.27 (s, 1, H-1''); 4.33–4.29 (m, 1, H-2'); 4.23–4.17 (m, 3, H-2'', 3', 4'); 4.08–3.96 (m, 3, H-3'', 5', 5'); 3.89–3.87 (m, 1, H-4''); 3.79 (2×d, 1, H-5''); 3.58 (2×d, 1, H-5''); 1.12–1.01 (m, 28, TIPDSi).
**2d**: yield 87%; ^1^H-NMR (CDCl_3_): δ (ppm) 7.89 (d, 1, H-6); 5.71 (d, 1, H-5); 5.64 (s, 1, H-1'); 5.39 (s, 1, H-1''); 4.53 (m, 1, H-2'); 4.30–4.19 (m, 3, H-2'', 3', 4'); 4.12–3.94 (m, 3, H-3'', 5', 5'); 3.76–3.71 (m, 1, H-4''); 3.66 (2×d, 1, H-5''); 3.36 (2×d, 1, H-5''); 1.11–1.02 (m, 28, TIPDSi).

### 3.3. General Procedure for the Synthesis of N-Protected-3',5'-O-(tetraisopropyldisiloxane-1,3-diyl)-2'-O-(2″,3″-di-O-benzoyl-β-d-ribofuranosyl) Ribonucleosides **3**

#### Transient Protecting Procedure

Compound **2** (2.5 g) was dried by co-evaporation with anhydrous pyridine and dissolved in dry pyridine (20 mL) and next the trimethylchlorosilane (1.94 g) was added. After 1 h benzoyl chloride (1.12 g) (in case of guanosine isobutyryl chloride 0.96 g) was added. The mixture was stirred for 2 h, cooled, and water (2 mL) was added. After 10 min a 29% aqueous solution of ammonia (3 mL) was added and the mixture was stirred at room temperature for 10 min. The reaction was then evaporated to a gum and partitioned between dichloromethane (100 mL) and a saturated aqueous solution of NaHCO_3_ (100 mL). The organic layers were evaporated to dryness and the residue was purified by flash chromatography on silica gel.

N-protected nucleosides were dried by threefold evaporation with anhydrous pyridine and suspended in dry pyridine (25 mL), and next the 4,4'-dimethoxytrityl chloride (1.3 g) was added. After 2 h TLC showed that the reaction was completed. The mixture was then evaporated to a gum and partitioned between dichloromethane (100 mL) and a saturated aqueous solution of NaHCO_3_ (100 mL). The organic layers were evaporated to dryness.

The residue was dried three times by evaporation with anhydrous pyridine and suspended in dry pyridine (25 mL) and next benzoyl chloride (1.05 g) was added. The reaction mixture was cooled to 5 °C. After 3 h TLC showed that the reaction was completed. The mixture was then evaporated to a gum and partitioned between dichloromethane (100 mL) and a saturated aqueous solution of NaHCO_3_ (100 mL). The organic layers were collected, combined and evaporated to dryness.

Next the residue was suspended in dichloromethane (3 mL) and a 10% solution of dichloroacetic acid in dichloromethane (10 mL) was added. After 10 min methyl alcohol (1 mL) was added. The TLC analysis showed that the reaction was completed. The mixture was partitioned between dichloromethane (100 mL) and a saturated aqueous solution of NaHCO_3_ (100 mL). The organic layer was evaporated to dryness and the residue was put on top of a silica gel column and purified by chromatography in a gradient of methanol (0%–2%) in dichloromethane. The pure product was obtained in the solid form.

**3a**: yield 75%; ^1^H-NMR (CDCl_3_): δ (ppm) 8.67 (s, 1, H-2), 8.45 (s, 1, H-8), 7.98–7.84 and 7.60–7.33 (m, 15, 3×Bz), 6.22 (s, 1, H-1'), 6.05 (m, 1, H-3''), 5.85 (d, 1, H-2''), 5.63 (s, 1, H-1''), 5.03 (m, 1, H-3'), 4.60–4.49 (m, 3, H-5'', 4″, 2'), 4.32–4.25 (m, 2, H-5'', 4'), 4.11–4.03 (m, 2, H-5', 5''-OH), 3.84–3.77 (m, 1, H-5'), 1.09–0.96 (m, 28, TIPDSi).
**3b**: yield 84%; ^1^H-NMR (CDCl_3_): δ (ppm) 8.36 (d, 1, H-6), 7.88–7.25 (m, 16, 3×Bz, H-5), 5.85 (m, 1, H-3''), 5.71 (s, 1, H-1'), 5.69 (d, 1, H-2''), 5.63 (s, 1, H-1″), 4.46 (m, 2, H-2', H-4'), 4.23–4.19 (m, 2, H-3', H-4''), 3.99–3.93 (m, 2, H-5'), 3.68–3.63 (m, 2, H-5''), 1.03–0.88 (m, 28, TIPDSi).
**3c**: yield 73%; ^1^H-NMR (CDCl_3_): δ (ppm) 12.20 (s, 1, N-H), 10.21 (s, 1, N-H), 8.16 (s, 1, H-8), 7.99–7.32 (m, 10, 2×Bz), 5.92 (s, 1, H-1'), 5.89 (t, 1, H-3''), 5.87 (s, 1, H-1''), 5.83 (d, 1, H-2''), 4.62 (m, 1, H-4''), 4.53 (t, 1, H-3'), 4.50 (s, 1, 5''-OH), 4.34 (d, 1, H-2'), 4.26–4.16 (m, 3, H-4'', 5'), 4.07–3.96 (4×d, 2, H-5''), 2.66 (m, 1, CH of *i*-bu), 1.28 (s, 3, CH_3_ of *i*-bu), 1.27 (s, 3, CH_3_ of *i*-bu), 1.10–0.95 (m, 28, TIPDSi).
**3d**: yield 81%; ^1^H-NMR (CDCl_3_): δ (ppm) 8.85 (s, 1, N-H), 7.77 (d, 1, H-6), 7.9–7.26 (m, 10, 2×Bz), 5.88 (m, 1, H-3''), 5.79 (d, 1, H-2''), 5.74 (s, H-1'), 5.72 (d, 1, H-5), 5.67 (s, 1, H-1''), 4.54 (m, 1, H-4''), 4.39 (d, 1, H-2'), 4.3–4.23 (m, 3, H-3', H-4', H-5'), 4.07–3.97 (m, 2, H-5', H-5''), 3.80 (2×d, 1, H-5''), 1.09–0.96 (m, 28, TIPDSi).

### 3.4. General Procedure for the Synthesis of N-Protected 3',5'-O-(tetraisopropyldisiloxane-1,3-diyl)-2'-O-(5″-H-phosphonate-2″,3″-di-O-benzoyl-β-d-ribofuranosyl) Ribonucleosides **4**

Compound **3** (2 g) was dried by threefold evaporation with anhydrous pyridine and suspended in a mixture of dry pyridine (10 mL) and dichloromethane (10 mL). Next the diphenyl phosphonate (1.19 g) was added. After 15 min the reaction was completed, which was shown in the TLC analysis. Next triethylamine (0.42 mL) and water (0.2 mL) were added. The reaction was evaporated to dryness and the residue was suspended in dichloromethane (20 mL). The organic solution was washed twofold with a portion of water (30 mL). The organic layer was evaporated to dryness and the residue was put on top of a silica gel column and purified by chromatography in a gradient of methanol (0%–5%) in dichloromethane with 1% triethylamine, which gave a pure product after lyophilization from benzene.

**4a**: yield 96%; ^31^P-NMR (D_2_O): δ = 3.01 ppm *J*_P-H_ = 610 Hz
**4b**: yield 98%; ^31^P-NMR (D_2_O): δ = 4.37 ppm *J*_P-H_ = 658 Hz
**4c**: yield 95%; ^31^P-NMR (D_2_O): δ = 4.87 ppm *J*_P-H_ = 640 Hz
**4d**: yield 96%; ^31^P-NMR (D_2_O): δ = 5.06 ppm *J*_P-H_ = 674 Hz

### 3.5. General Procedure for the Synthesis of 5″Phosphate 2'-O-β-d-ribofuranosyl Ribonucleosides **5**

Compound **4** (0.51 g) was dried by threefold evaporation with anhydrous pyridine and dissolved in a mixture of dry pyridine (10 mL) and dichloromethane (2.5 mL). Next 3-hydroxypropionitrile (0.142 g) was added, and after 10 min pivaloyl chloride (0.06 g) was added. The ^31^P-NMR analysis was performed after 45 min.

**5a**: ^31^P-NMR (D_2_O): δ = 9.46 and 9.03 ppm *J*_P-H_ = 717 Hz and 7.19 Hz
**5b**: ^31^P-NMR (D_2_O): δ = 10.10 and 9.45 ppm *J*_P-H_ = 734 Hz
**5c**: ^31^P-NMR (D_2_O): δ = 11.66 and 9.56 ppm *J*_P-H_ = 742 Hz and 726 Hz
**5d**: ^31^P-NMR (D_2_O): δ = 9.49 and 9.43 ppm *J*_P-H_ = 720 Hz

After 20 min water (0.2 mL) and a 1 M solution of iodine in pyridine (1 mL) was added. The ^31^P-NMR analysis was performed after 10 min, and showed that the reaction was totally complete.

**6a**: ^31^P-NMR (D_2_O): δ = −0.63 ppm
**6b**: ^31^P-NMR (D_2_O): δ = −1.47 ppm
**6c**: ^31^P-NMR (D_2_O): δ = −1.34 ppm
**6d**: ^31^P-NMR (D_2_O): δ = −0.58 ppm

A small portion of ethanethiol (20 µL) was added to the solution. The reaction mixture was partitioned between dichloromethane (100 mL) and a saturated aqueous solution of NaHCO_3_ (100 mL). The organic layer was evaporated to dryness and dissolved in tetrahydrofuran (THF) (10 mL). Next a 1 M tetra-*n*-butylammonium fluoride (TBAF) in THF (3 mL) solution was added. When the TLC analysis showed that the reaction went to completion (2 h), the reaction mixture was evaporated to dryness. Next the residue was suspended in pyridine (5 mL), and concentrated aqueous ammonia (4 mL) was added. The reaction was kept at 55 °C overnight. Next, the reaction mixture was concentrated and traces of pyridine were removed by co-evaporation with toluene (3 × 15 mL). The residue was purified by RP C-18 column chromatography under reduced pressure in linear gradient acetonitrile (1%–20%) in water. After that the aqueous solution of the product was passed over a column with Sephadex CM-50, collected, combined and evaporated. The pure product was lyophilized from water.

**7a**: yield 89%; FAB-MS: *m/z* [M−H]^−^; Calcd for C_15_H_21_N_5_O_11_P^−^, 478.10; found *m/z* 477.9; Calcd for C_15_H_20_N_5_O_11_PNa^−^, 500.08; found *m/z* 499.8; ^31^P-NMR (D_2_O): δ =1.481ppm; ^1^H-NMR (D_2_O): δ (ppm) 8.24 (s, 1, H-2); 8.08 (d, 1, H-8); 6.06 (d, 1, H-1'); 4.80 (d, 1, H-1''); 4.74 (t, 1, H-2'); 4.43 (q, 1, H-3'); 4.12 (q, 1, H-4'); 4.06 (q, 1, H-2''); 4.01 and 3.99 (2×d, 1, H-3''); 3.77 (m, 1, H-4''); 3.73 (2×d, 1, H-5'); 3.68 (2×d, 1, H-5'); 3.53 (m, 1, H-5″); 3.37 (m, 1, H-5″); ^13^C-NMR (D_2_O): δ (ppm) 156.58 (s, C-6); 153.34 (s, C-2); 149.18 (s, C-4); 142.62 (s, C-8); 120.03 (s, C-5); 108.28 (s, C-1″); 88.49 (s, C-1'); 86.80 (s, C-4'); 82.73 (d, C4″); 80.42 (s, C-2'); 75.24 (s, C-2″); 71.61 (s, C-3″); 70.56 (s, C-3'); 65.90 (d, C-5″); 62.4 (s, C-5').
**7b:** yield 83%; FAB-MS: *m/z* [M–H]**^−^**; Calcd for C_14_H_21_N_3_O_12_P^−^ 454.09; found *m/z* 454.1; Calcd for C_14_H_20_N_3_O_12_PNa^−^, 476.07; found *m/z* 475.9; ^31^P-NMR (D_2_O): δ = 0.359 ppm; ^1^H-NMR (D_2_O): δ (ppm) 7.82 (d, 1, H-6); 6.07 (d, 1, H-5); 5.94 (d, 1, H-1'); 5.13 (s, 1, H-1″); 4.45 (t, 1, H-2'); 4.35 (t, 1, H-3'); 4.24 (q, 1, H-3''); 4.17 (d, 1, H-2''); 4.08 (m, 1, H-4''); 4.08 (m, 1, H-4'); 3.92 (m, 1, H-5''); 3.87 (2×d, 1, H-5'); 3.76 (2×d, 1, H-5'); 3.74 (m, 1, H-5''); ^13^C-NMR (D_2_O): δ (ppm) 167.02 (s, C-4); 158.07 (s, C-2); 144.19 (s, C-6); 108.31 (s, C-1''); 97.36 (s, C-5); 91.42 (s, C-1'); 85.03 (s, C-4'); 82.68 (d, C-4''); 79.95 (s, C-2'); 75.19 (s, C-2''); 71.69 (s, C-3''); 69.33 (s, C-3'); 66.57 (s, C-5''); 61.85 (s, C-5'').
**7c**: yield 82%; FAB-MS: *m/z* [M−H]**^−^**; Calcd for C_15_H_21_N_5_O_12_P^−^ 494.09; found *m/z* 493.8; Calcd for C_15_H_20_N_5_O_12_PNa^−^ 516.07; found *m/z* 515.7; ^31^P-NMR (D_2_O): δ = 3.115 ppm; ^1^H-NMR (D_2_O): δ (ppm) 7.88 (d, 1, H-8); 5.93 (d, 1, H-1'); 4.90 (d, 1, H-1''); 4.62 (t, 1, H-2'); 4.40 (q, 1, H-3'); 4.11 (q, 1, H-4'); 4.03 (q, 1, H-2''); 4.02 (d, 1, H-3''); 3.87 (m, 1, H-4''); 3.73 (2×d, 1, H-5'); 3.67 (2×d, 1, H-5'); 3.61 (m, 1, H-5''); 3.52 (m, 1, H-5''); ^13^C-NMR (D_2_O): δ (ppm) 159.86 (s, C-6); 154.62 (s, C-2); 152.06 (s, C-4); 139.71 (s, C-8); 117.46 (s, C-5); 108.53 (s, C-1''); 88.04 (s, C-1'); 85.66 (s, C-4'); 82.94 (d, C4''); 80.34 (s, C-2'); 75.28 (s, C-2''); 71.90 (s, C-3''); 70.17 (s, C-3'); 65.78 (d, C-5''); 61.98 (s, C-5').
**7d**: yield 81%; FAB-MS: *m/z* [M−H]**^−^**; Calcd for C_14_H_20_N_2_O_13_P^−^ 455.07; found *m/z* 454.9; Calcd for C_14_H_19_N_2_O_13_PNa^−^ 477.05; found *m/z* 476.8; ^31^P-NMR (D_2_O): δ = 2.176 ppm; ^1^H-NMR (D_2_O): δ (ppm) 7.73 (d, 1, H-6); 5.74 (d, 1, H-5); 5.82 (d, 1, H-1'); 5.98 (s, 1, H-1''); 4.31 (t, 1, H-2'); 4.21 (t, 1, H-3'); 4.14 (q, 1, H-3''); 4.04 (d, 1, H-2″); 3.95 (m, 1, H-4″''); 3.91 (m, 1, H-4'); 3.76 (m, 1, H-5''); 3.72 (2×d, 1, H-5'); 3.67 (2×d, 1, H-5'); 3.55 (m, 1, H-5''); ^13^C-NMR (D_2_O): δ (ppm) 167.18 (s, C-4); 152.26 (s, C-2); 144.26 (s, C-6); 108.84 (s, C-1''); 103.12 (s, C-5); 91.05 (s, C-1'); 84.83 (s, C-4'); 82.94 (d, C-4''); 80.49 (s, C-2'); 75.25 (s, C-2''); 71.70 (s, C-3''); 69.33 (s, C-3'); 66.04 (d, C-5''); 61.57 (s, C-5').

### 3.6. Synthesis of 6-N-Benzoyl-2'-O-(5″-H-phosphonate-2″,3″-di-O-benzoyl-β-d-ribofuranosyl)-adenosine (**8*a***) and 2-N-Isobutyryl-2'-O-(5″-H-phosphonate-2″,3″-di-O-benzoyl-β-d-ribofuranosyl)-guanosine (**8*c***)

Compounds **4a** or **4c** (0.457 g) were dissolved in tetrahydrofuran (1 mL) and 1 M TBAF in THF (1.2 mL) was added. After 2 h the TLC analysis showed that the reaction was completed. Then the reaction mixture was partitioned between dichloromethane (100 mL) and a saturated aqueous solution of NaHCO_3_ (100 mL). The organic layer was dried with anhydrous Na_2_SO_4_ and the residue was put on top of a silica gel column and purified by chromatography in a gradient of methanol (0%–8%) in dichloromethane and 1% Et_3_N. The pure product was obtained as a foam by evaporation.

**8a**: yield 89%; ^31^P-NMR (D_2_O): δ 3.01 ppm, *J*_P-H_ = 611 Hz; ^1^H-NMR (CDCl_3_): δ (ppm) 8.93 (s, H-2); 8.58 (d, H-8); 7.2–7.9 (m, 15H, arom.) 6.80 (d, H-1'); 4.80 (d, H-1''); 4.74 (t, H-2'); 4.43 (q, H-3'); 4.12 (q, H-4'); 4.06 (q, H-2″); 4.01 and 3.99 (2×d, H-3''); 3.77 (m, H-4''); 3.73 (2×d, H-5'); 3.68 (2×d, H-5'); 3.53 (m, H-5''); 3.37 (m, H−5'').
**8c**: yield 84%; ^31^P-NMR (D_2_O): δ = 2.855 ppm, *J*_P-H_ = 609 Hz; ^1^H-NMR (CDCl_3_): δ (ppm) 13.25 (s, 1, N-H); 12.38 (s, 1, N-H); 7.71 (s, 1, H-8); 7.92–7.87 (m, 2, arom.); 7.68–7.26 (m, 8, arom.); 5.89 (d, 1, H-1'); 5.73 (m, 1, H-2''); 5.69 (s, 1, H-1''); 5.65 (m, 1, H-3″); 4.69 (m, 1, H-4''); 4.40 (m, 1, H-3'); 4.28–4.21 (m, 2, H-2', 5''); 4.13–4.04 (m, 2, H-4', 5''); 3.56–3.49 (m, 2, H-5', 5'); 3.20 (s, 3, CH_3_-ibu); 3.17 (m, 1, CH of i-bu); 3.14 (s, 3, CH_3 _of i-bu).

### 3.7. Synthesis of 6-N-benzoyl-5'-O-(4,4'-dimethoxytrityl)-2'-O-(5″-H-phosphonate-2″,3″-di-O-benzoyl-β-d-ribofuranosyl)adenosine (**9*a***) and 2-N-isobutyryl-5'-O-(4,4'-dimethoxytrityl)-2'-O-(5″-H-phosphonate-2″,3″-di-O-benzoyl-β-d-ribofuranosyl)guanosine (**9*c***)

Compounds **8a** or **8c** (0.31 g) were dried by threefold evaporation with anhydrous pyridine and suspended in dry pyridine (3 mL). Next the 4,4'-dimethoxytrityl chloride (0.14 g) was added. After 3 h the TLC analysis showed that the reaction was completed. Then the reaction mixture was partitioned between dichloromethane (25 mL) and a saturated aqueous solution of NaHCO_3_ (50 mL). The organic layer was dried with anhydrous Na_2_SO_4_ and the residue was put on top of a silica gel column and purified by chromatography in a gradient of methanol (0%–8%) in dichloromethane with constant 1% Et_3_N. The pure product was evaporated with toluene and lyophilized from benzene. The final compound was obtained as the corresponding triethylamine salt.

**9a**: yield 93%; FAB-MS: *m/z* [M−H]**^−^**; Calcd for C_57_H_51_N_5_O_15_P^−^, 1076.31; found *m/z* 1076.1; ^31^P-NMR (CDCl_3_): δ = 4.02 ppm *J*_P-H_ = 629 Hz; ^1^H-NMR (CDCl_3_): δ (ppm) 8.75 (s,1,H-2); 8.59 (s, 1, H-8); 7.9–7.3 (m, 28, H-arom.); 5.92 (s, 1, H-1'); 5.82 (t, 1, H-3''); 5.66 (2×d, 1, H-2''); 5.12 (d, H-1″); 5.09 (m, 1, H-3'); 4.62 (m, 1, H-2') 4.39 (d, 1, H-5'); 4.29 (d, 1, H-5'); 4.22–4.05 (m, 3, H-4', 5'', 4''); 3.76 (s,6,O-CH_3_); 3.52 (2×d, 2, CH_2_); 1.31 (t, 3, CH_3_).
**9c**: yield 91%; ^31^P-NMR (CDCl_3_): δ = 1.458 ppm *J*_P-H_ = 603 Hz; ^1^H-NMR (CDCl_3_): δ (ppm) 13.60 (s, 1, N-H); 12.38 (s, 1, N-H); 7.82 (s, 1, H-8); 7.97–7.85 (m, 4, arom); 7.53–7.15 (m, 15, H-arom.); 6.82–6.76 (m, 4, H-arom); 6.42 (d, 1, H-1'); 5.92 (s, 1, H-1''); 5.82 (t, 1, H-3''); 5.67 (m, 1, H-2''); 5.33 (m, 1, H-4''); 4.81 (m, 1, H-3'); 4.58 (d, 1, H-2'); 4.53 (m, 1, H-4'); 4.41 (2×d, 1, H-5'); 4.17–4.12 (m, 3, H-5', 5'', 5″); 3.76 (s, 6, CH_3_) 2.34 (m, 1, CH of ibu); 1.19 (s, 3, CH_3_ of ibu); 1.17 (s, 3, CH_3_ of ibu).

### 3.8. Synthesis of 6-N-Benzoyl-5'-O-(4,4'-dimethoxytrityl)-2'-O-(5″-dicyanoethyl phosphotriester 2″,3″-di-O-benzoyl-β-d-ribofuranosyl)adenosine (**11*a***) and 2-N-isobutyryl-5'-O-(4,4'-dimethoxy-trityl)-2'-O-(5″-dicyanoethyl phosphotriester 2″,3″-di-O-benzoyl-β-d-ribofuranosyl)guanosine (**11*c***)

Compounds **9a** or **9c** (110 mg) were dissolved in a mixture of pyridine and dichloromethane (3:7). 3-hydroxypropionitrile (0.02 g) was then added to the stirred reaction mixture. After 10 min pivaloyl chloride (13 mg) was added. The reaction was controlled by ^31^P-NMR analysis.

**10a**: ^31^P-NMR (Py): δ = 9.351 ppm *J*_P-H_ = 719 Hz and δ = 9.153 ppm *J*_P-H_ = 720 Hz
**10c**: ^31^P-NMR (Py): δ = 9.748 ppm *J*_P-H_ = 726 Hz and δ = 9.428 ppm *J*_P-H_ = 720 Hz

When the reaction was completed, the next portion of 3-hydroxypropionitrile (120 mg) was added. 1 M I_2_ in dry pyridine (0.2 mL) was added to the stirring reaction. The reaction was completed in 10 min (^31^P-NMR analysis). After 30 min ethanethiol (0.10 mL) was added and the solution was evaporated. The residue was partitioned between dichloromethane with 1% pyridine (25 mL) and phosphate buffer pH 7 (50 mL). The organic layer was evaporated and the residue was put on top of a silica gel column and purified by chromatography in a gradient of methanol (0%–1%) in dichloromethane. The final product was lyophilized from benzene.

**11a**: yield 42%; ^31^P-NMR (CDCl_3_): δ = −2.29 ppm; ^1^H-NMR (CDCl_3_): δ (ppm) 8.69 (s, 1, H-2); 8.36 (s, 1, H-8); 8.1–7.26 (m, 28, arom); 6.88 (2×d, 4, trytyl-arom) 6.39 (d, 1, H-1'); 5.78 (t, 1, H-3''); 5.68 (2×d, 1, H-2''); 5.42 (d, H-1''); 5.25 (t, 1, H-3'); 4.70 (t, 1, H-2') 4.47 (m, 1, H-4'); 4.37–4.25 (m, 6, H-5', 4'', CH_2_); 4.17 (m, 1, H-5') 3.79 (s, 6, O-CH_3_); 3.52 (2×d, 1, H-5''); 3.44 (2×d, 1, H-5''); 2.79 (m, 4, CH_2_CN).
**11c**: yield 37%; ^31^P-NMR (CDCl_3_): δ = −2.031 ppm; ^1^H-NMR (CDCl_3_): δ (ppm) 12.20 (s, 1, N-H); 10.25 (s, 1, N-H); 7.74 (s, 1, H-8); 7.96–7.85 (m, 4, arom); 7.58–7.21 (m, 15, arom); 6.85–6.79 (m, 4, arom); 6.32 (d, 1, H-1'); 5.89 (t, 1, H-3''); 5.30 (s, 1, H-1''); 5.70 (m, 1, H-2″); 4.71 (t, 1, H-3'); 4.57 (m, 1, H-4''); 4.55 (d, 1, H-2'); 4.48 (m, 1, H-4'); 4.46 (2×d, 1, CH_2_); 4.46–4.39 (m,3, H-5', 5', 5'', 5''); 3.77 (s, 6, CH_3_) 2.34 (m, 1, CH of *i*-bu); 1.19 (s, 3, CH_3_ of *i*-bu); 1.17 (s, 3, CH_3_ of i-bu).

### 3.9. Synthesis of 6-N-Benzoyl-3'-[(2-O-cyanoethyl)-N,N-diisopropylphosphoramidite]-5'-O-(4,4'-dimethoxytrityl)-2'-O-(5″-dicyanoethyl phosphotriester 2″,3″-di-O-benzoyl-β-d-ribofuranosyl)-adenosine (**12*a***) and 2-N-Isobutyryl-3'-[(2-O-cyanoethyl)-N,N-diisopropylphosphoramidite]-5'-O-(4,4'-dimethoxytrityl)-2'-O-(5″-dicyanoethyl phosphotriester 2″,3″-di-O-benzoyl-β-d-ribofuranosyl)-guanosine (**12*c***)

First **11a** and **11c** (60 mg) and 1-*H*-tetrazole (3.5 mg) as solid were dried under reduced pressure for 12 h. Next **11a** and **11c** was dissolved in dry dichloromethane. Bis(diisopropylamino)(2-cyanoethoxy)phosphine (17 mg) was added to the mixture under argon atmosphere. Next 1-H-tetrazole (3.5 mg) was added in small portions. The TLC analysis showed that the phosphitylation was completed in 4 h. The reaction mixture was put on top of a silica gel column and purified by chromatography in a gradient of ethyl acetate (0%–60%) in hexane with constant content of triethylamine (10% by vol.). Collected fractions were combined and evaporated. The pure product was obtained after lyophilization from benzene.

**12a**: yield 45%;^31^P-NMR (benzene): δ (ppm) = −2.48, −2.42, 149.97, 150.58; FAB-MS: *m/z* [M−H]**^−^**; Calcd for C_72_H_76_N_9_O_17_P_2_^+^, 1400.48; found *m/z* 1401.4
**12c**: yield 42%; ^31^P-NMR (benzene): δ (ppm) = −3.09, 149.93, 14970.

### 3.10. Synthesis of Dimers ArT and Ar(p)T

A reactor for oligonucleotide synthesis was filled with a solid support charged with thymidine. The reactor was closed with a filter and placed in a DNA/RNA automatic synthesizer. The standard procedure involved removal of the dimethoxytrityl group from the support by means of dichloroacetic acid and absorption of the dimethoxytrityl cation was measured. After the support in the reactor was washed with acetonitrile, the reactor was opened, and the support was placed in a tube with dry acetonitrile. A solution of 0.5 M of 5-(ethylthio)-1-*H*-tetrazole in anhydrous acetonitrile and solutions of 3'-phosphoramidites: **12a** and 5'-*O*-dimethoxytrityl-2'-*O*-(tri-2,3,5-*O*-benzoylribosyl-1)adenosine 3'-phosphoramidite obtained according to ref [8] were dissolved in anhydrous acetonitrile in separate tubes.

3 Å molecular sieves were added to the prepared solutions and left in sealed vessels for 2 h. Next, the above described solutions of 3'-phosphoramidites were added to the relevant tubes containing the deprotected support. After 2 min 150 μL of a 5-(ethylthio)-1-*H*-tetrazole solution were added with a syringe. Condensation was performed for 12 min, and the tube was gently rocked periodically. After that the entire mixture was centrifuged, and the solution from above the support was decanted. Each support was washed three times with 2 mL of acetonitrile and placed in the reactor columns again. Then, each reactor was placed in a DNA/RNA automatic synthesizer and oxidized with an iodine solution. After the reactor was washed, the support was treated with dichloroacetic acid. After deprotection the reactor was treated with a concentrated aqueous solution of ammonia. The solution was placed in a sealed vessel and left for 24 h at 55 °C. Then the liquid was evaporated down, and the residue was dissolved in 0.40 mL of water and placed on a column filled with Sephadex C-25 gel. Three fractions of 1.5 mL were collected, combined and evaporated. FAB MS was performed for negative and positive ions.

*Ar(p)T* (**D1**); FAB-MS: *m/z* [M−H]**^−^**; Calcd for C_25_H_34_O_18_N_7_P_2_, 782.14; found *m/z* 782.0.
*ArT* (**D2**); FAB-MS: *m/z* [M–H]**^−^**; Calcd for C_25_H_33_O_15_N_7_P, 702.17; found *m/z* 702.2.

## 4. Conclusions

A new approach to the chemical synthesis of 5''-phosphates of 2'-*O*-ribosylnucleosides via *H*-phosphonate chemistry was described. This approach allowed us to obtain the appropriate 3'-phosphoramidite building blocks that for the first time allowed the synthesis of a dinucleoside monophosphate with a fully natural unit. This will be the platform for introduction of 5''-phosphates 2'-*O*-ribosylnucleoside 5''-phosphate units into oligonucleotides in the future.

## Supplementary Materials

Supplementary materials can be accessed at: http://www.mdpi.com/1420-3049/18/12/14780/s1.
