# Supplementary material for: Novel Method of Synthesis of 5''-Phosphate 2'-O-ribosyl-ribonucleosides and Their 3'-Phosphoramidites
Source: Molecules. 2013 Nov 29;18(12):14780–96. doi: 10.3390/molecules181214780 (PMC6269823; doi:10.3390/molecules181214780)

Supplementary Materials

Figure S1. 1H-NMR of Compound **7a**.

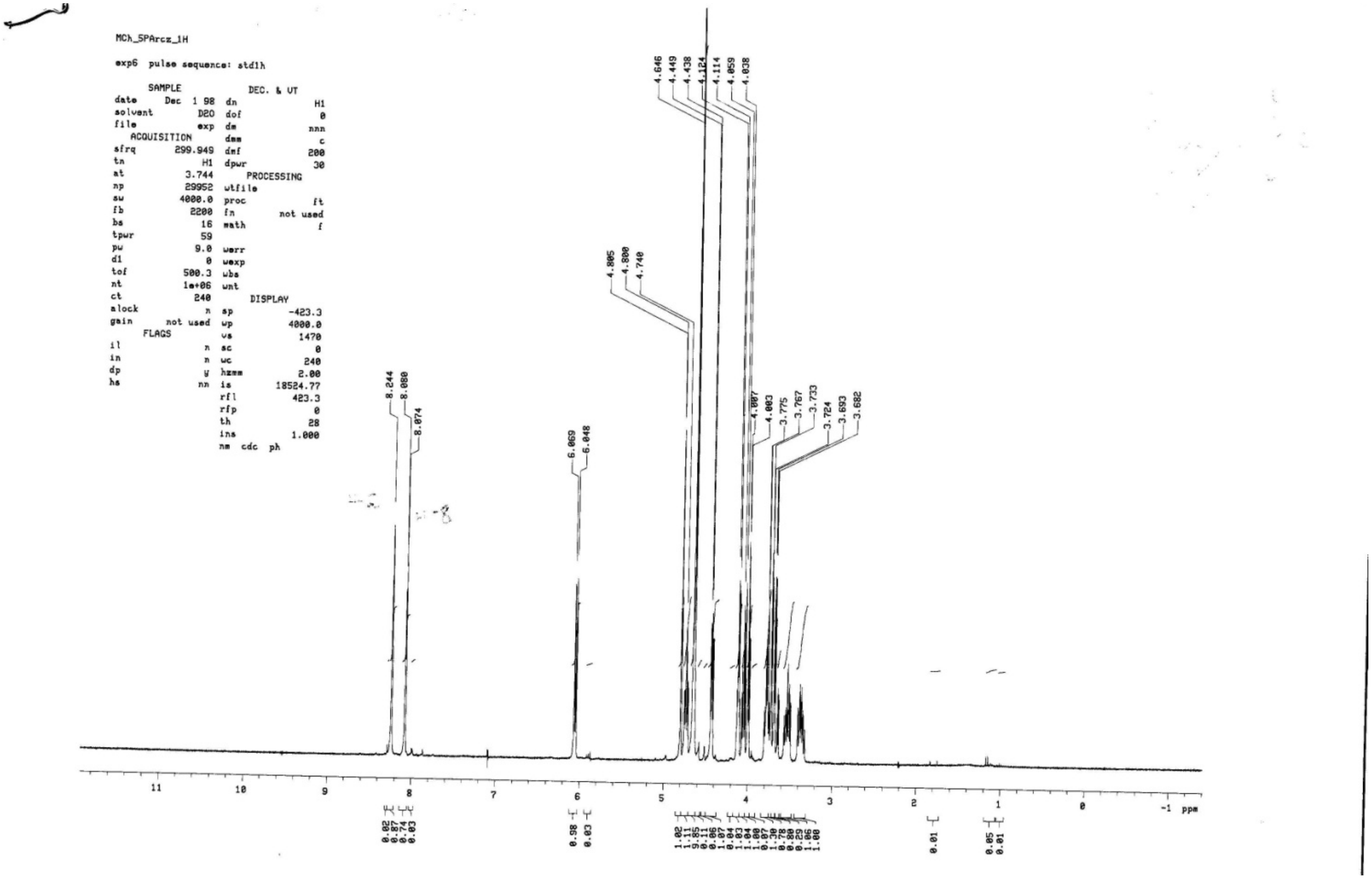

Figure S2. ^{13}C-NMR of Compound **7a**.

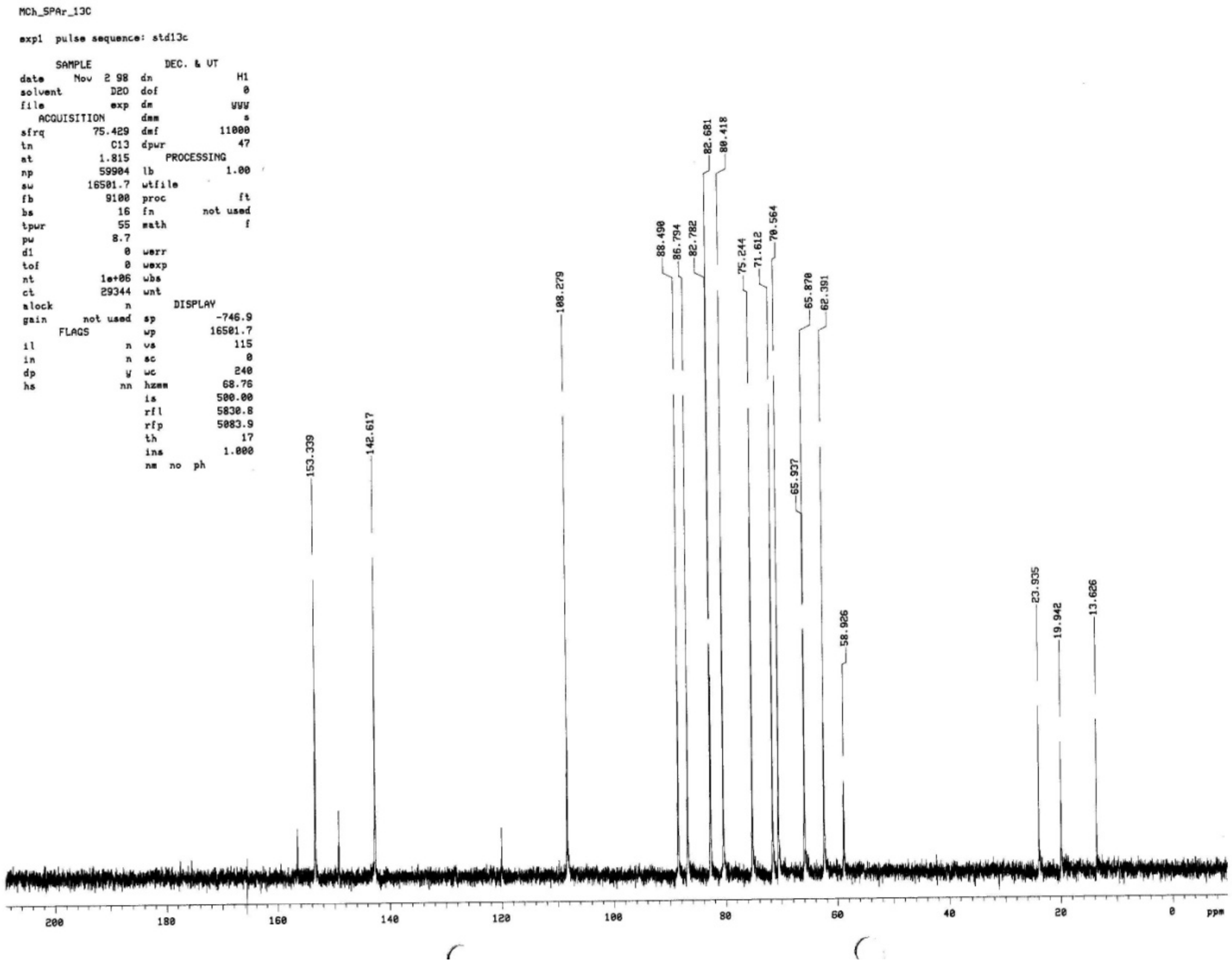

Figure S3. ^{31}P-NMR of Compound **7a**.

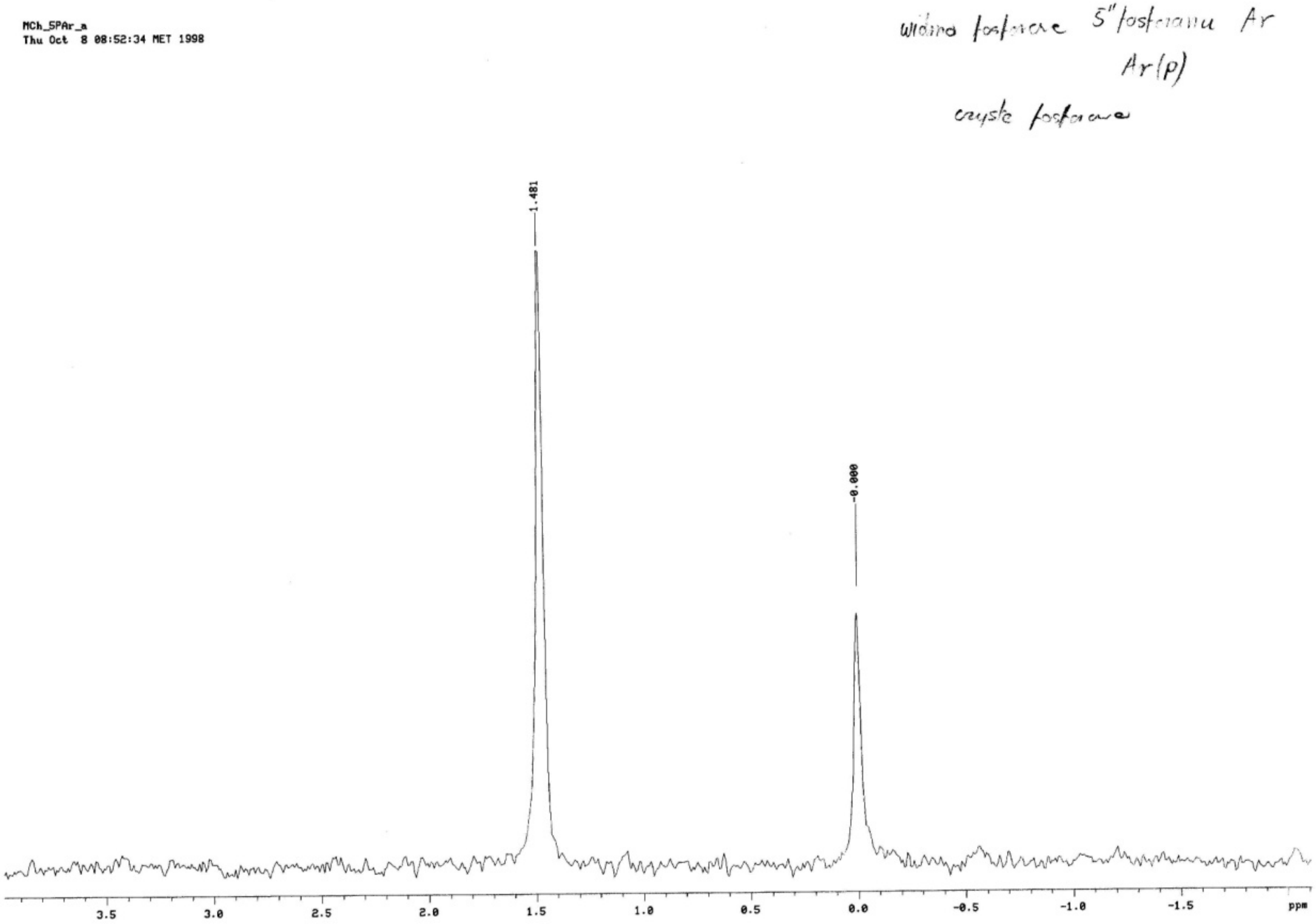

Figure S4. MS Analysis 1 of Compound **7a.**

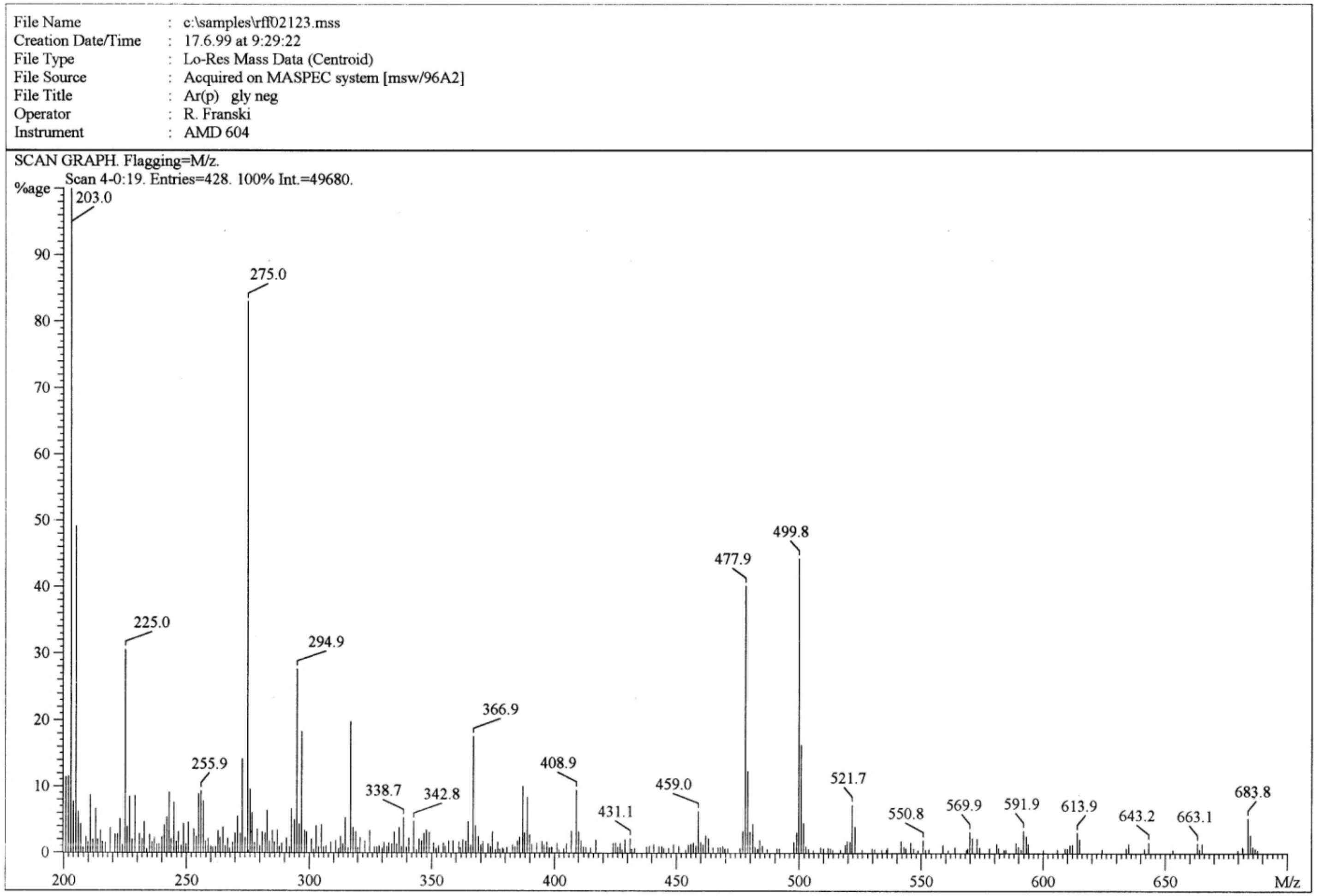

Figure S5. MS Analysis 2 of Compound **7a.**

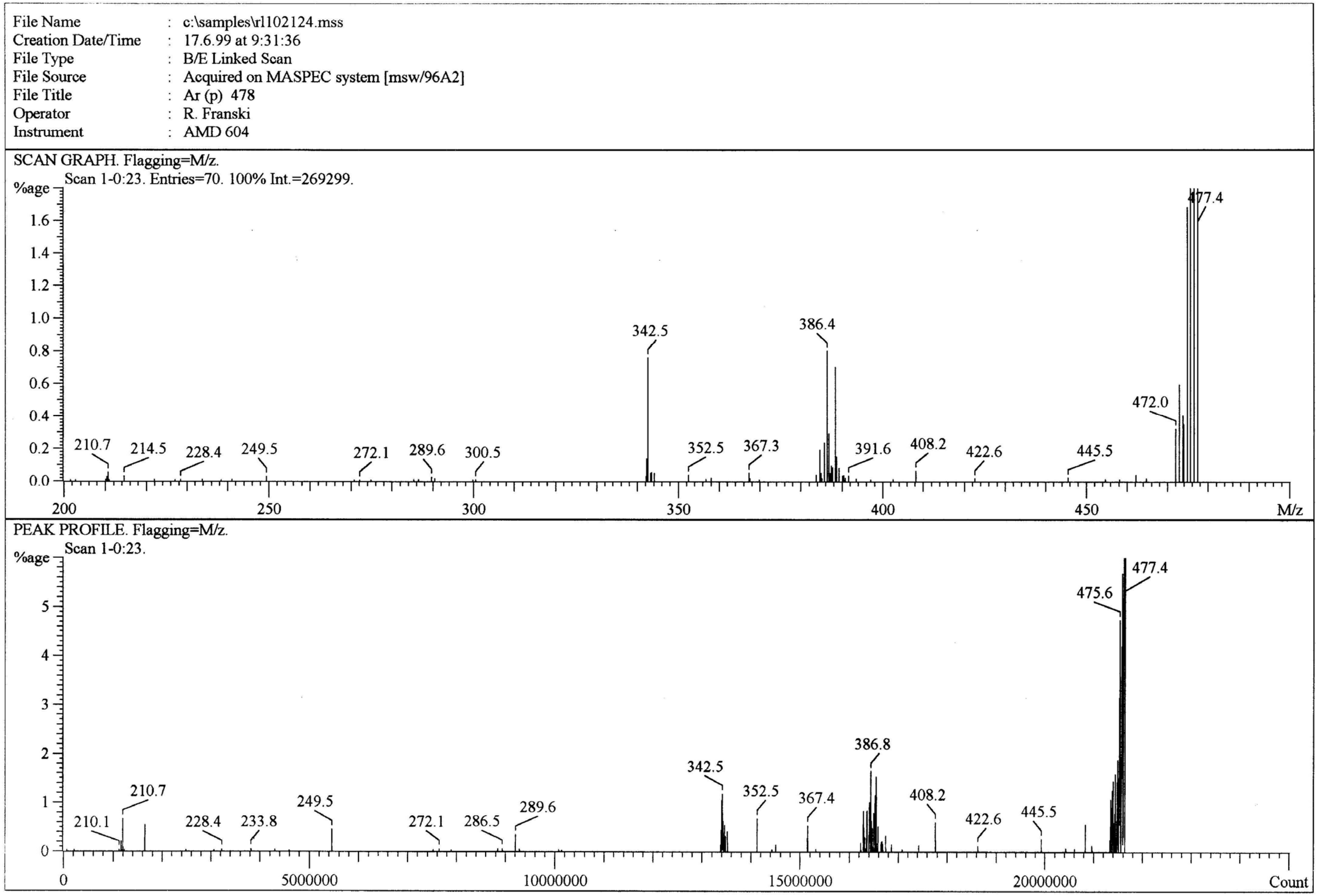

Figure S6. [1] H-NMR of Compound 7b.

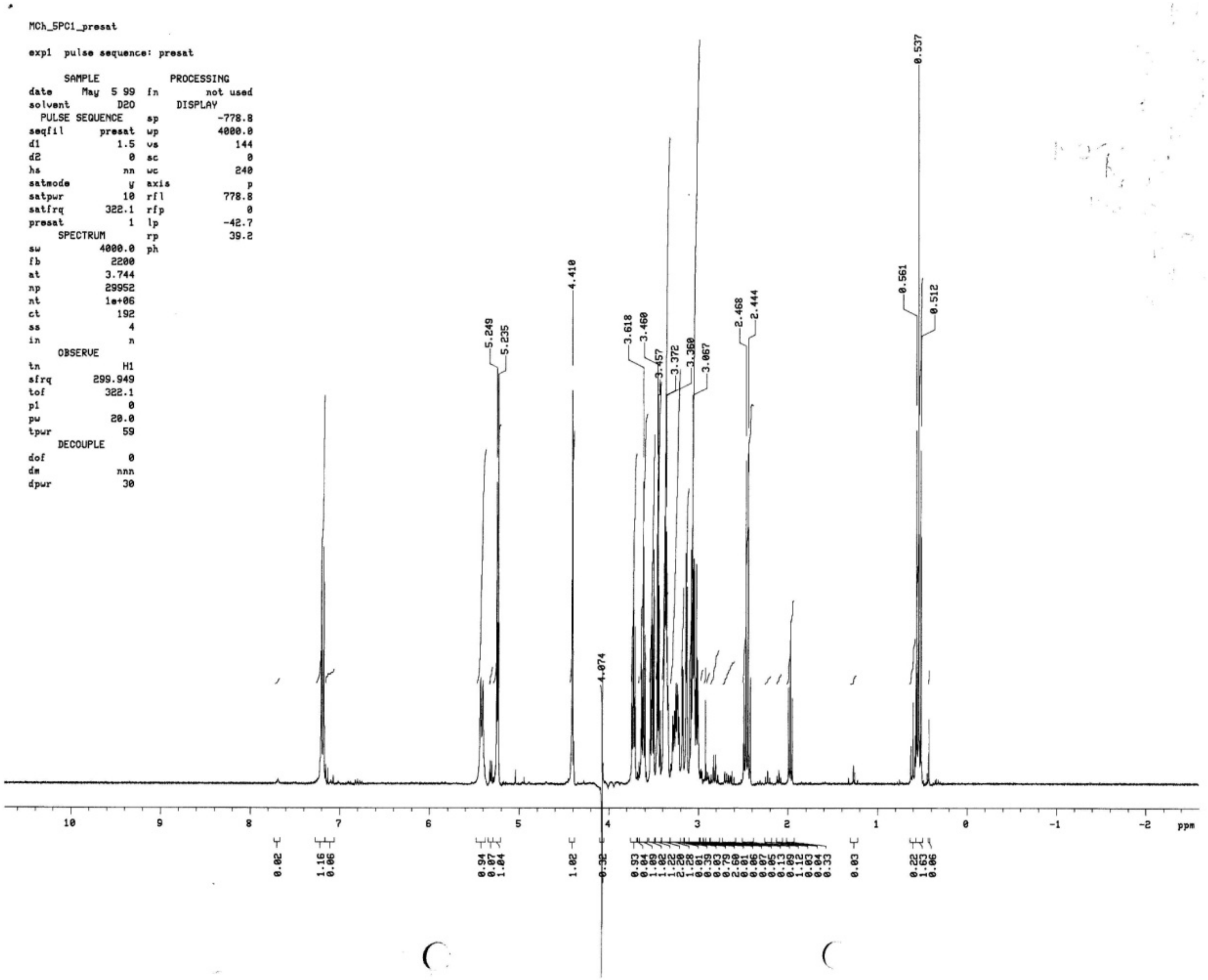

Figure S7. ^{13}C-NMR of Compound **7b**.

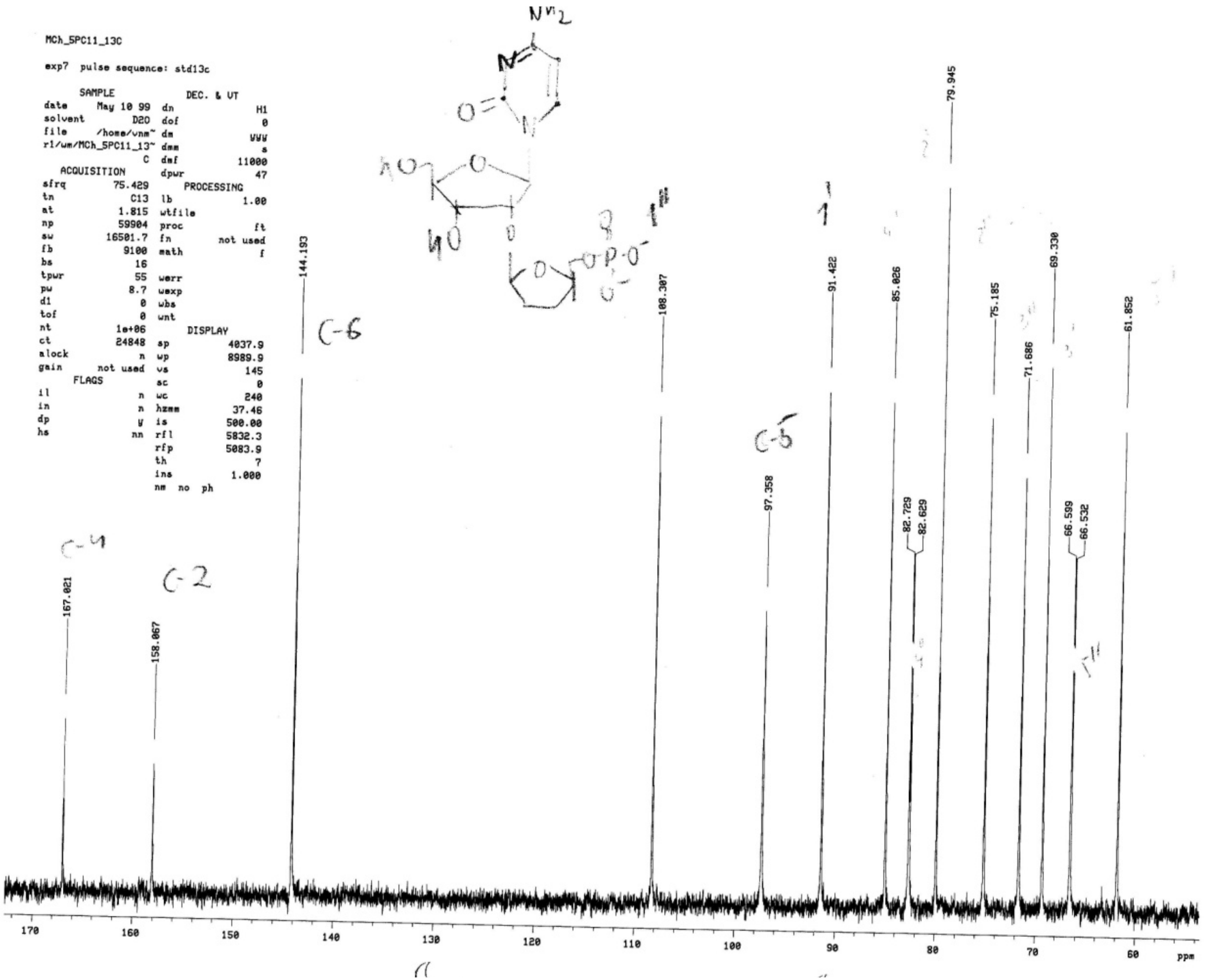

Figure S8. ^{31}P-NMR of Compound **7b**.

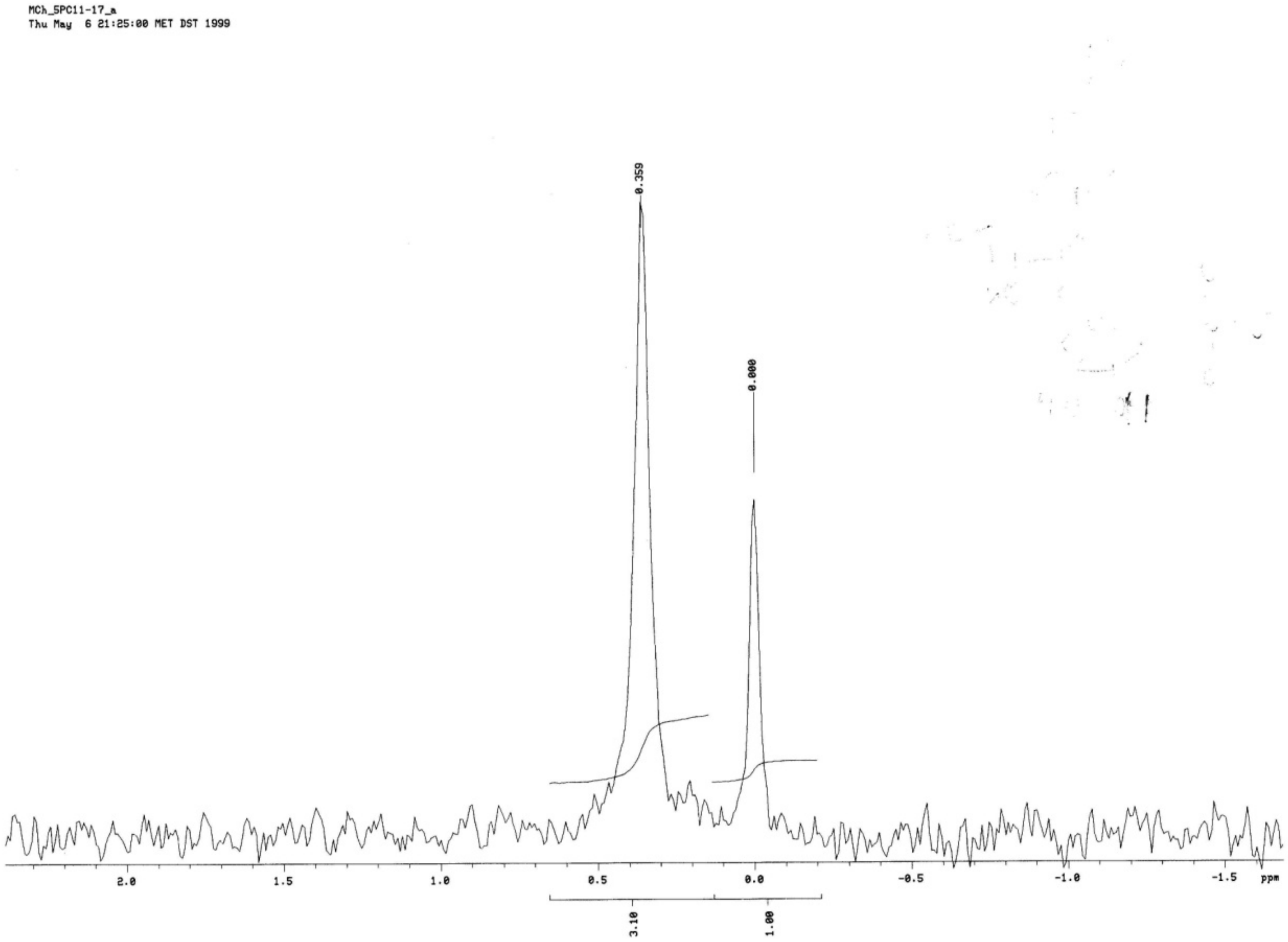

Figure S9. MS Analysis 1 of Compound **7b**.

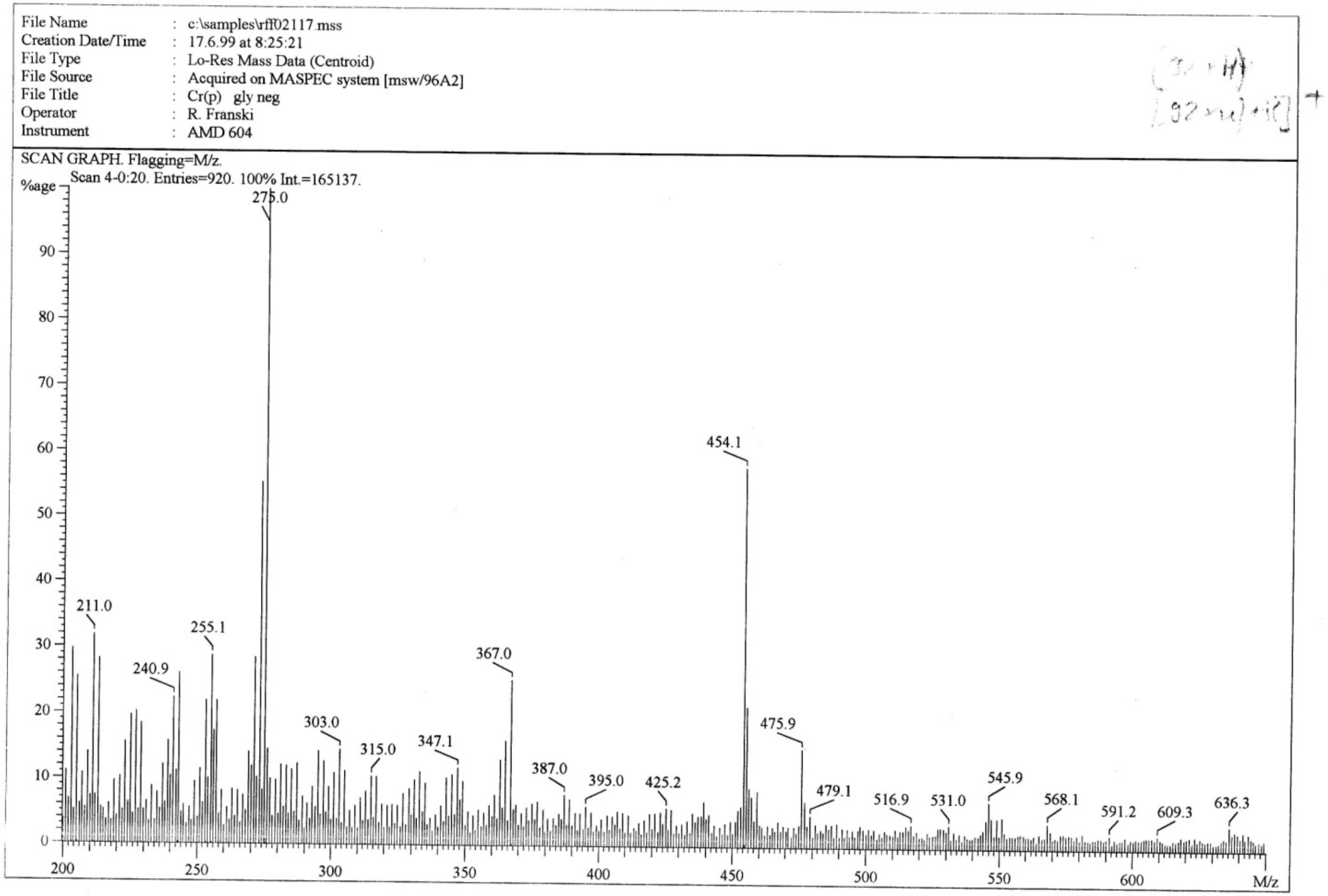

Figure S10. MS Analysis 2 of Compound **7b**.

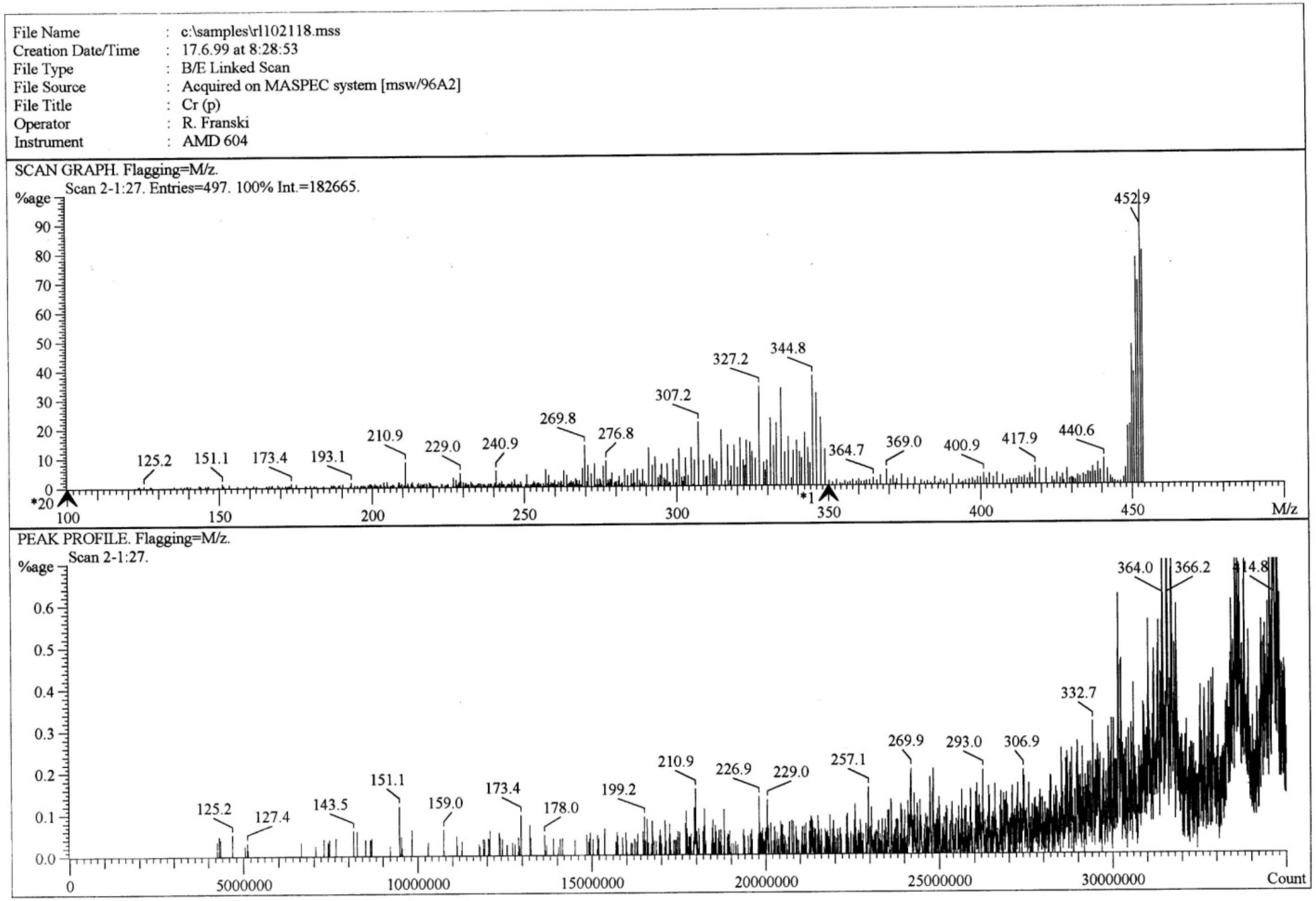

Figure S11. ^{1}H-NMR of Compound **7c.**

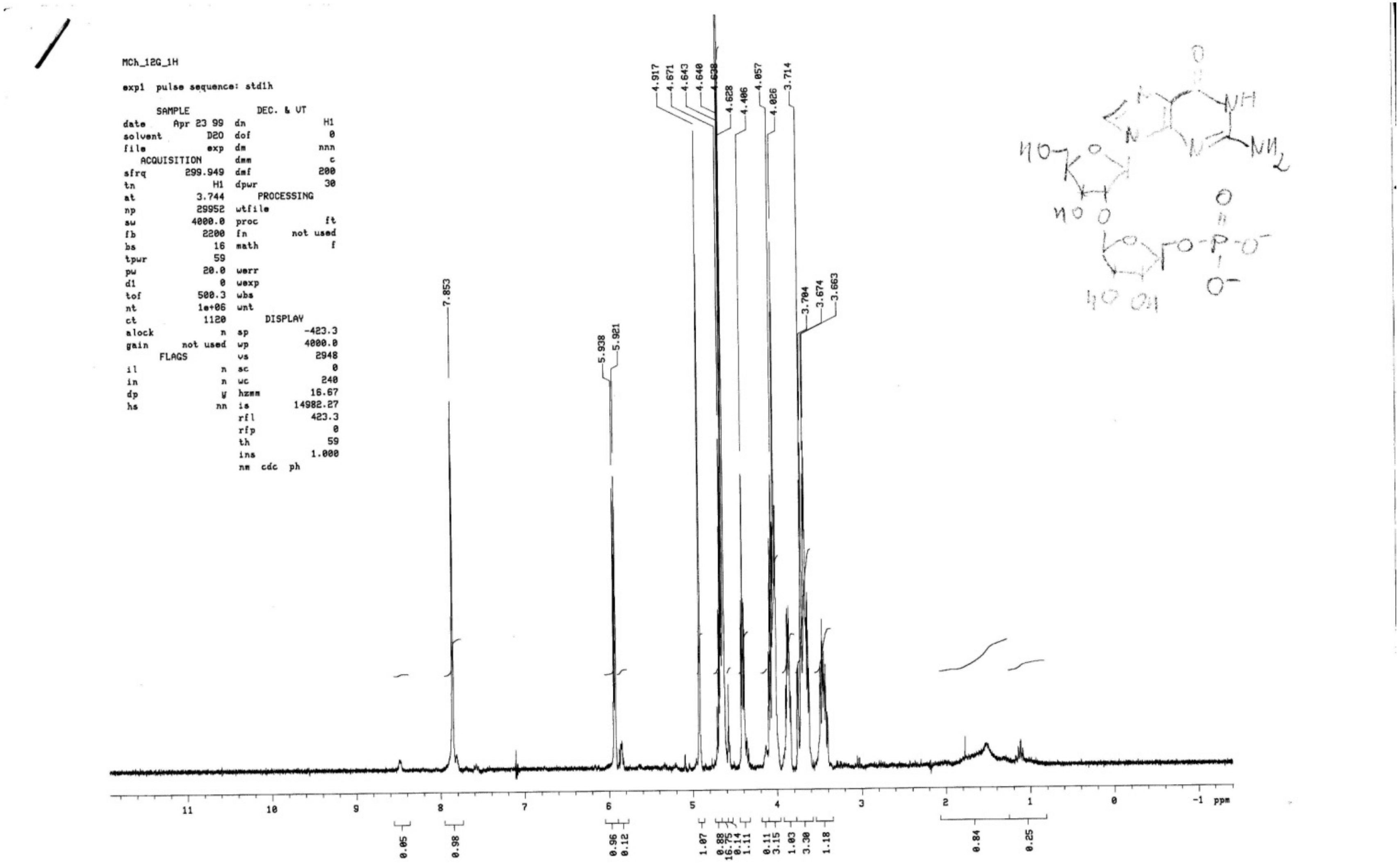

Figure S12. ^{13}C-NMR of Compound **7c.**

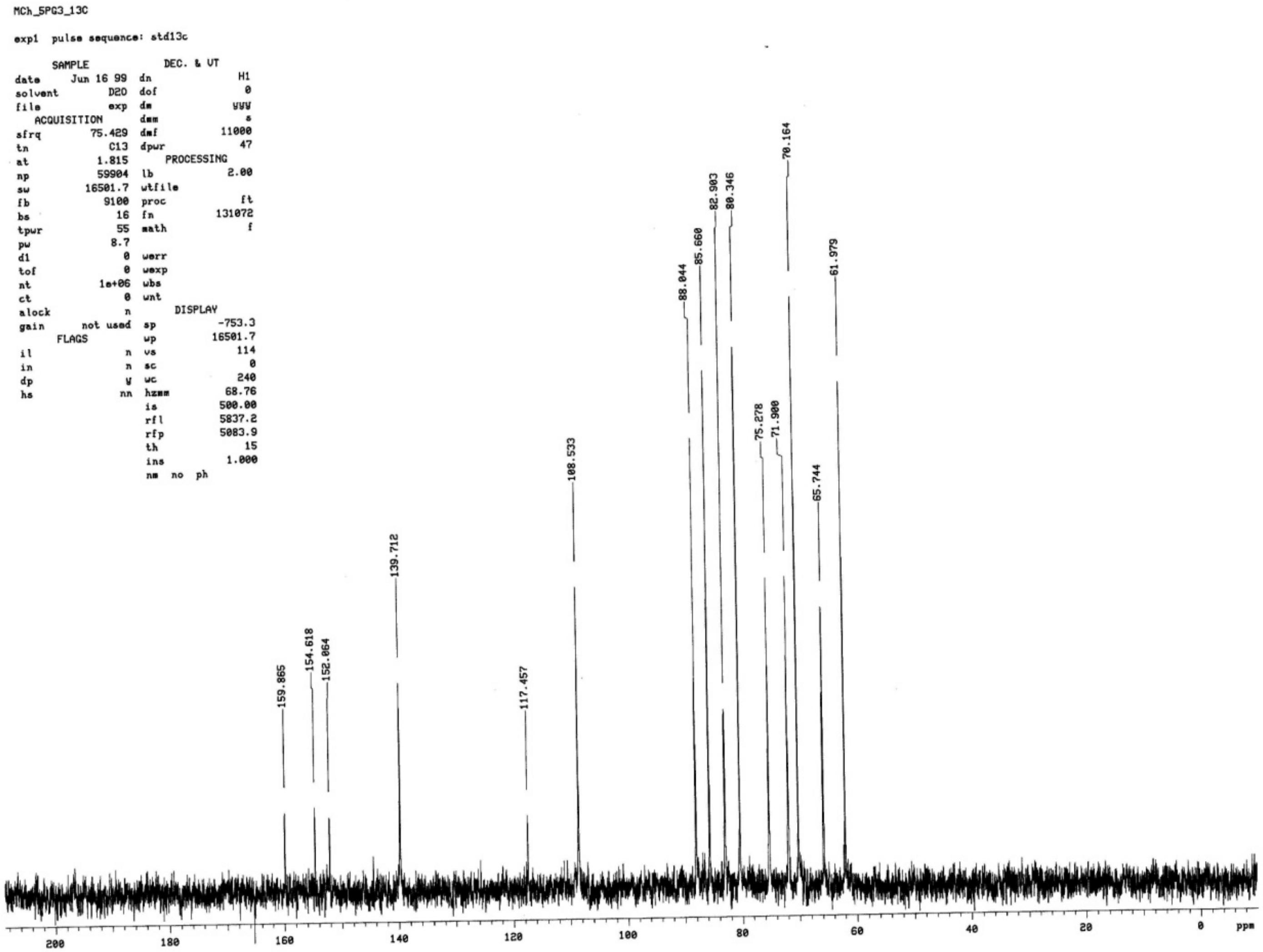

Figure S13. ^{31}P-NMR of Compound **7c.**

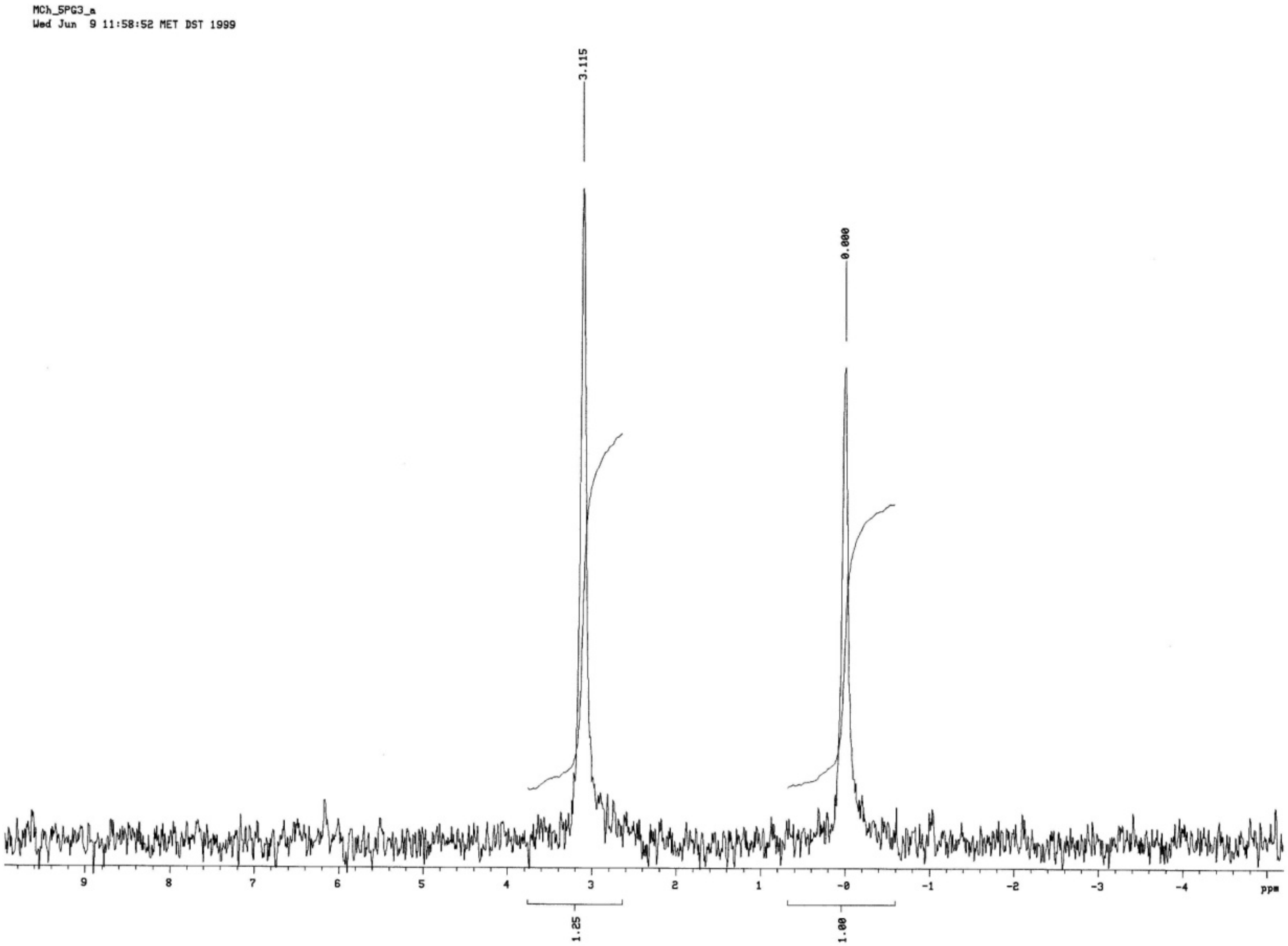

Figure S14. MS Analysis 1 of Compound **7c.**

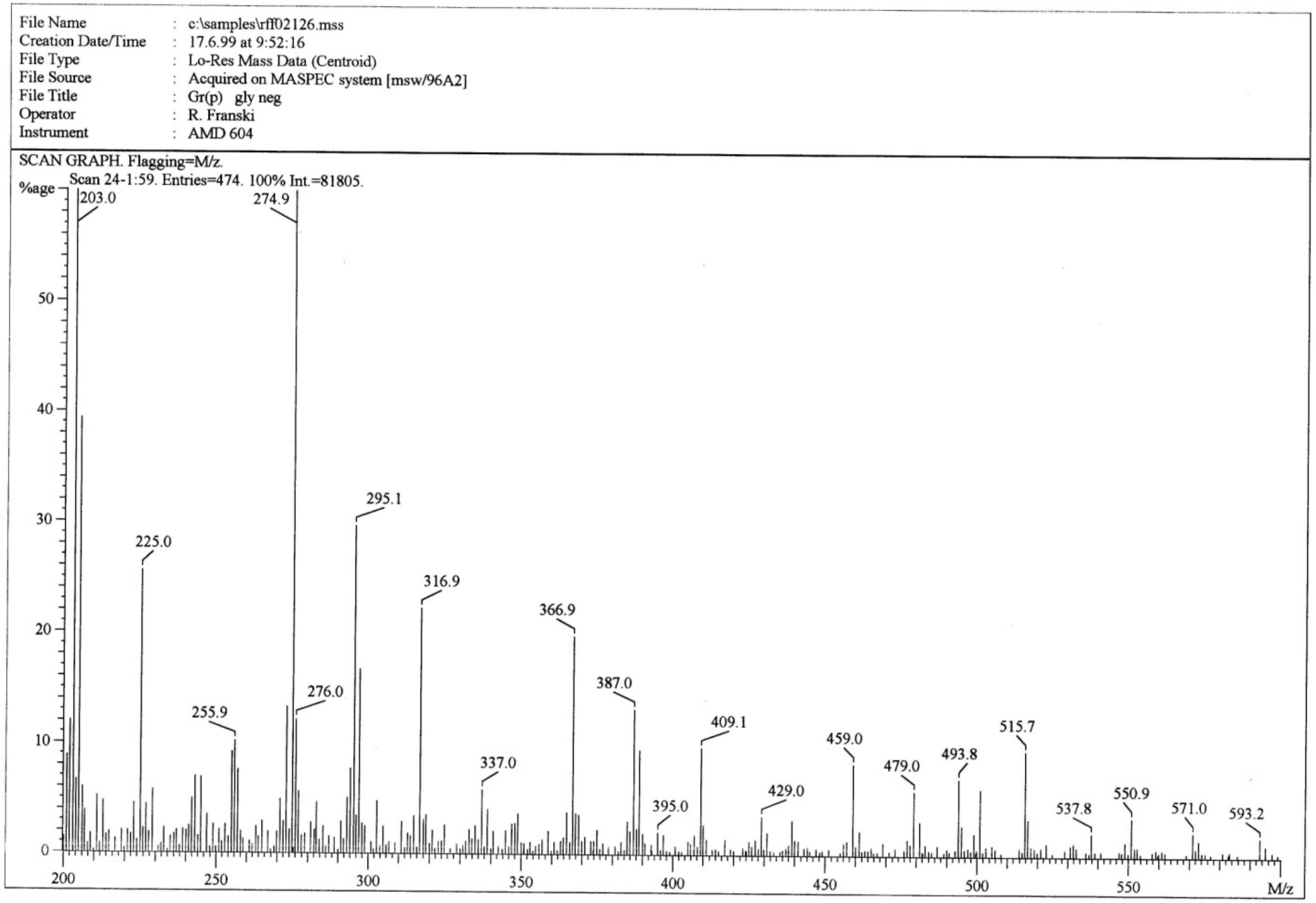

Figure S15. MS Analysis 2 of Compound **7c.**

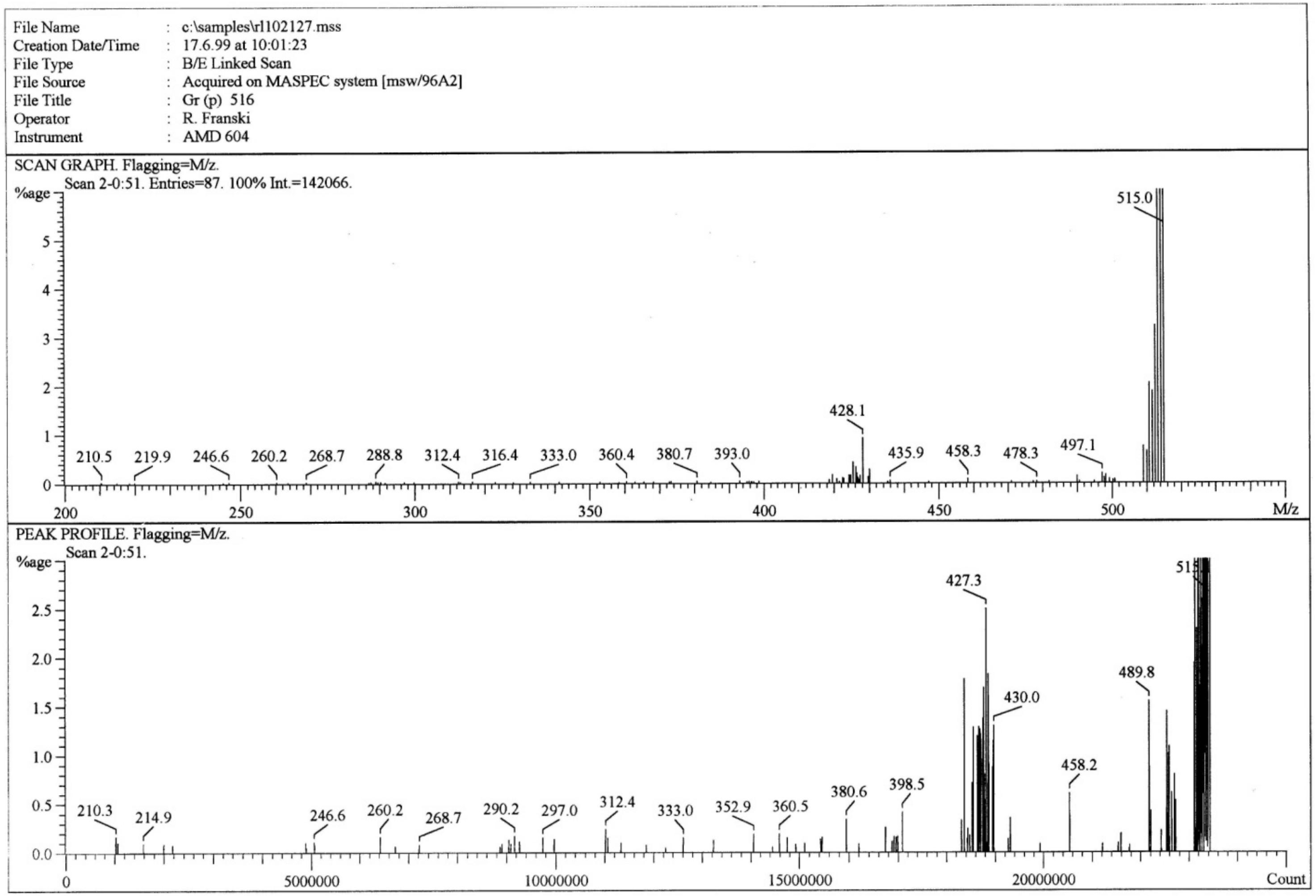

Figure S16. Atomic composition of Compound **7c.**

ASD01828.TXT

ATOMIC COMPOSITION REPORT

```
File Name        : c:\samples\test1.mss.
File Date/Time   : 17.6.99 at 10:17:11
File Type        : Hi-Res Mass Spectrum
File Source      : Acquired on MASPEC system [msw/96A2]
                   as c:\samples\rfv02128.mss
File Title       : Gr (p)
Source File      : c:\samples\rfv02128.mss
Source Scan(s)   : 8:64

Sort Field       : M/z (ascending).

Scan Filter      : none.
```

Selected isotopes:

Symbol	Min	Max	V'cy	Name
C	9	18	4	Carbon-12
H	10	23	1	Hydrogen-1
N	0	5	3	Nitrogen-14
O	0	14	2	Oxygen-16
Na	0	1	1	Sodium-23
P	0	1	3	Phosphorous-31

Allowable error = minimum of 10.0 ppm, 5.0 mmu.

Number of Peaks=31.
Base Peak=516.06013, 100% Int.=6716.

Mass	(%)	Calculated	ppm	mmu	Formula
494.09273	58.64	494.09244	-0.6	-0.3	C15.H21.N5.O12.P
516.07396	99.34	516.07440	0.9	0.4	C15.H20.N5.O12.Na.P

***** End of Atomic Composition Report *****

Figure S17. ^{1}H-NMR of Compound 7d.

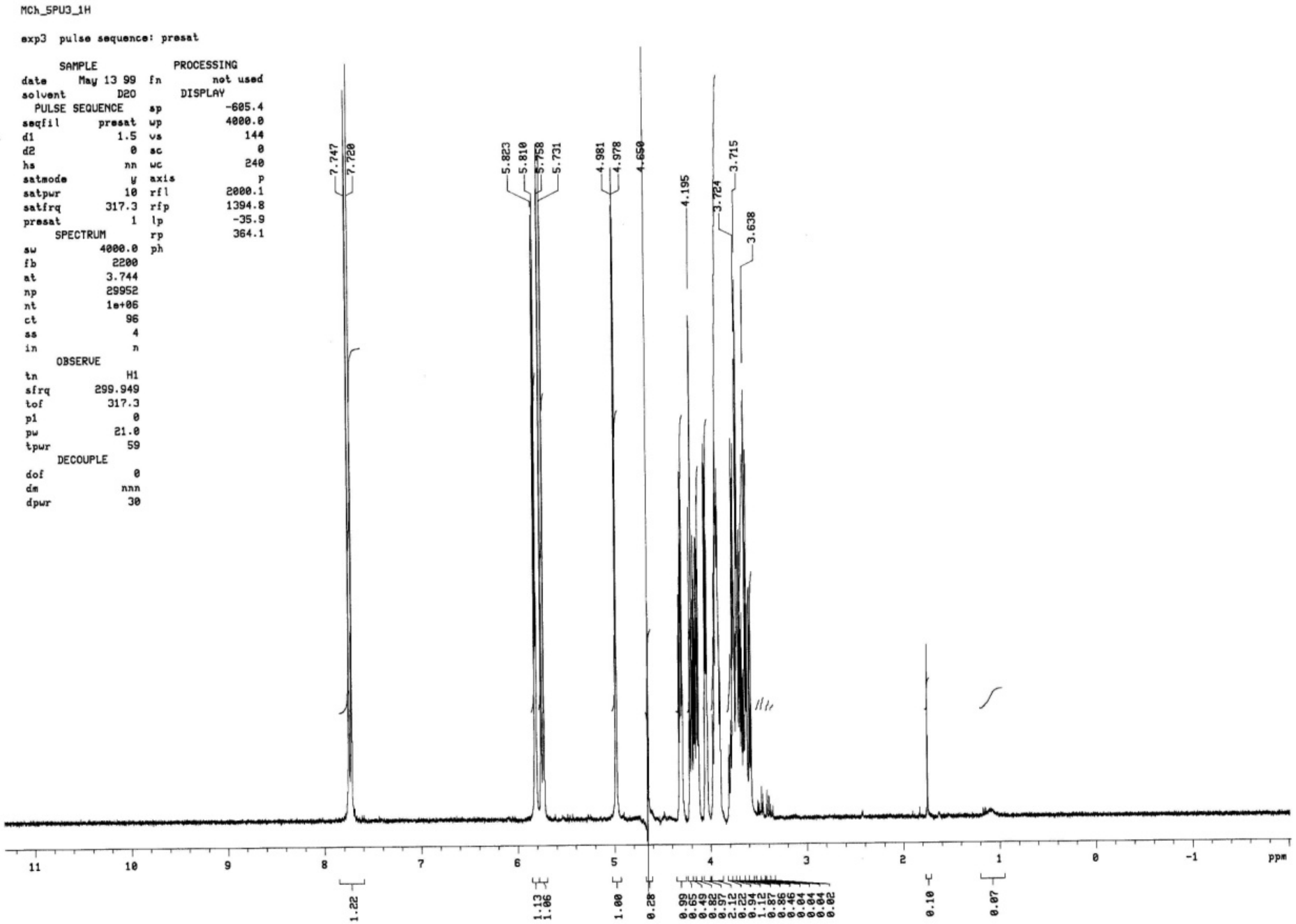

Figure S18. ^{13}C-NMR of Compound **7d**.

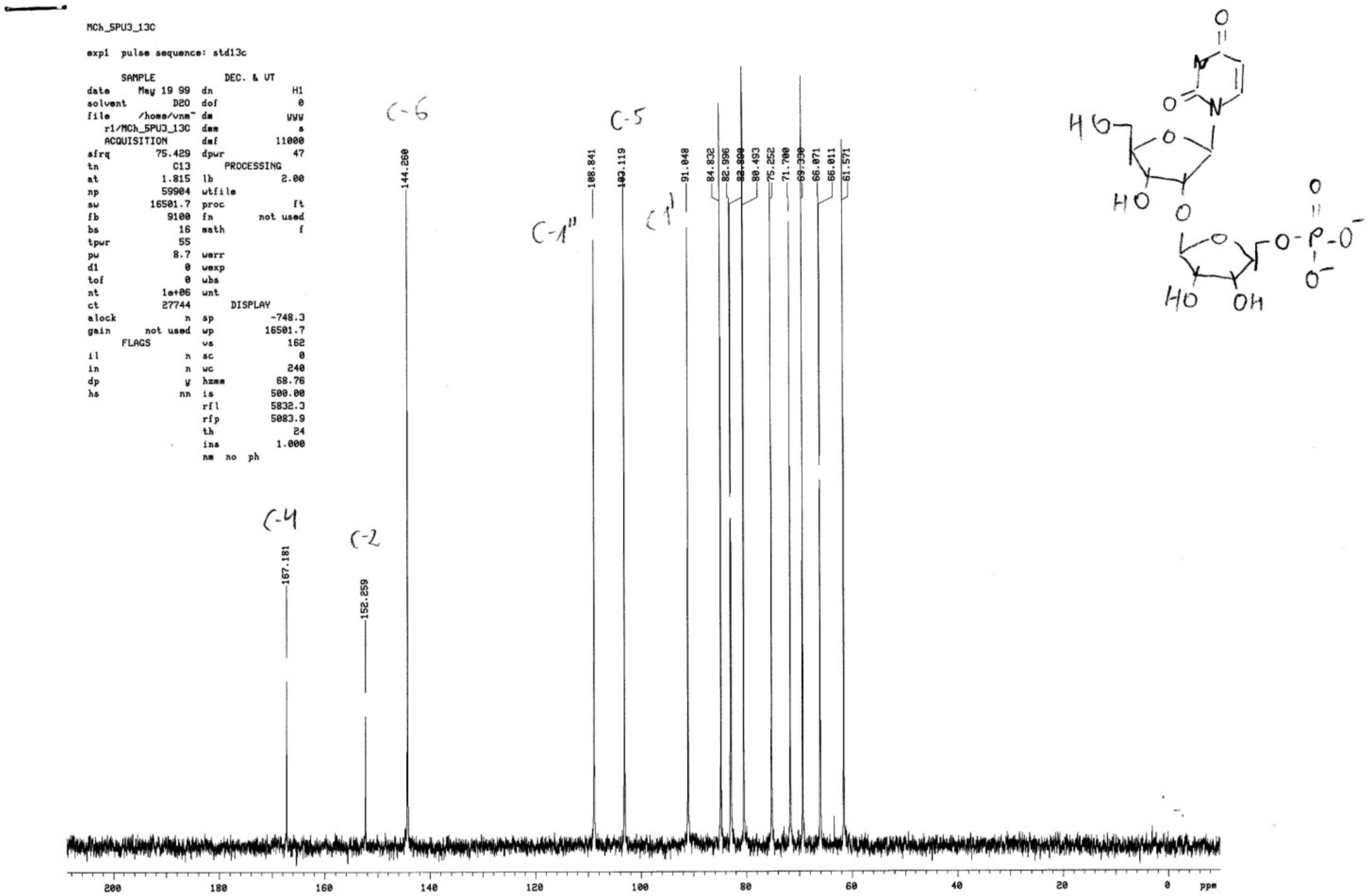

Figure S19. ^{31}P-NMR of Compound **7d.**

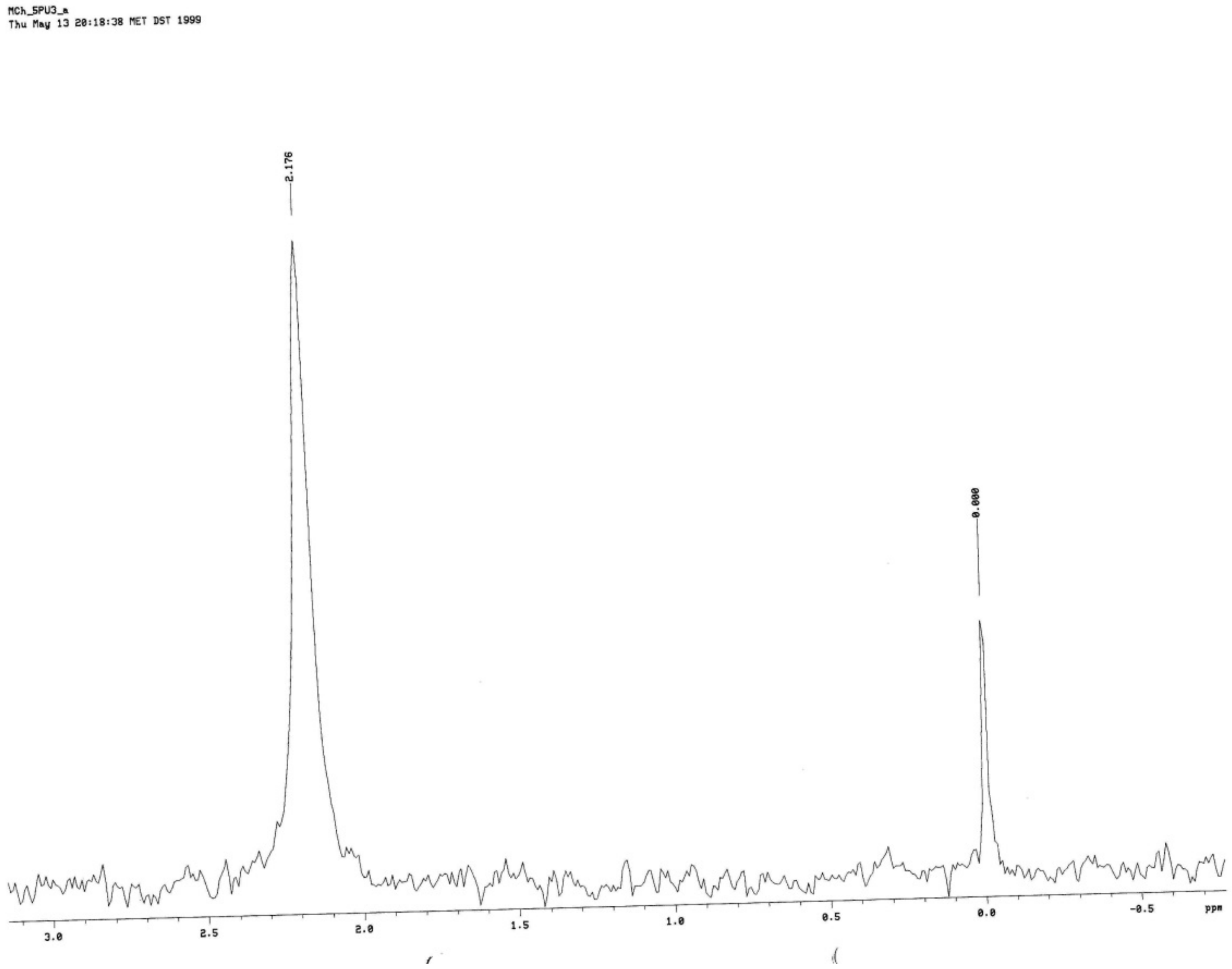

Figure S20. MS Analysis 1 of Compound **7d.**

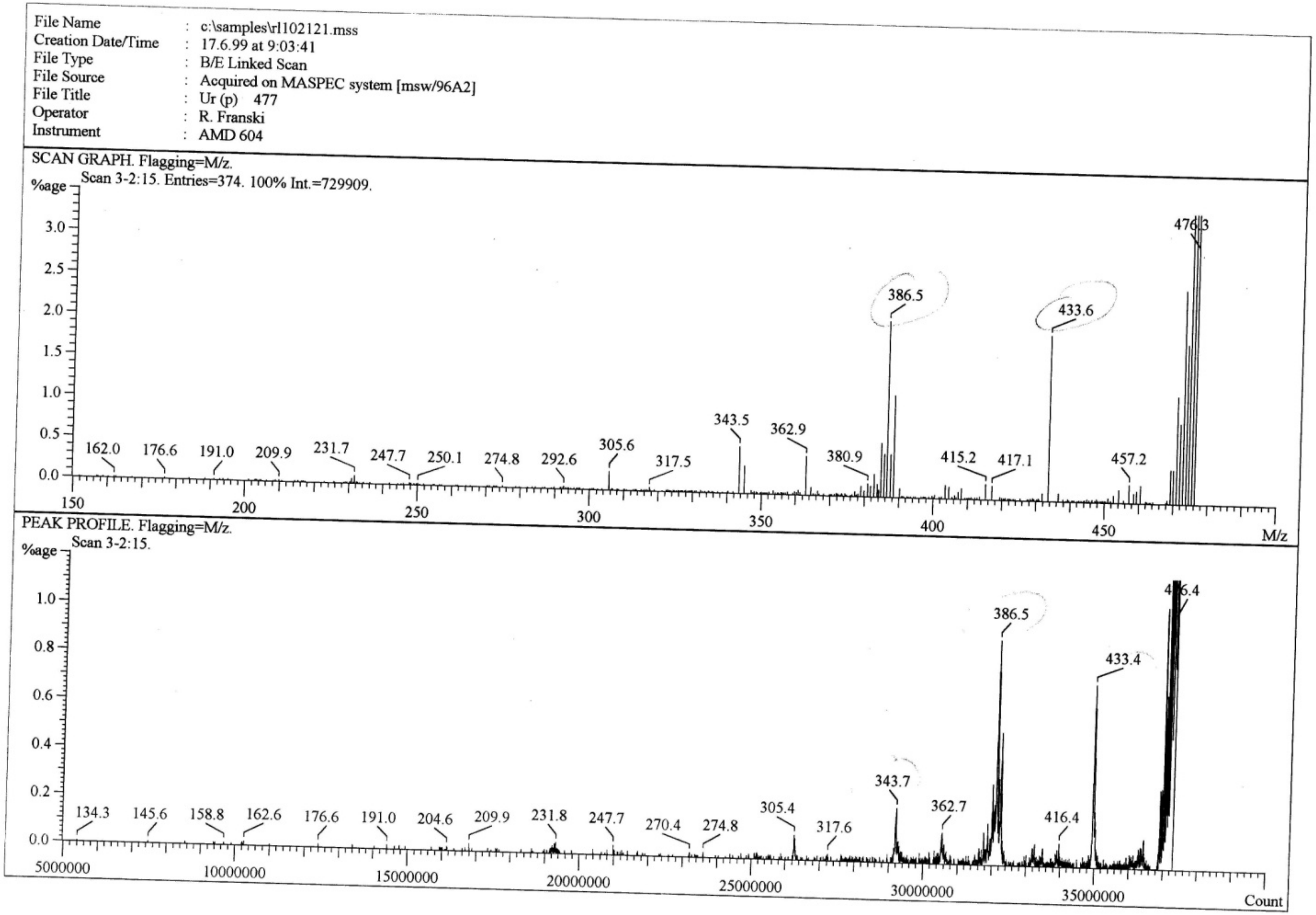

Figure S21. MS Analysis 2 of Compound **7d.**

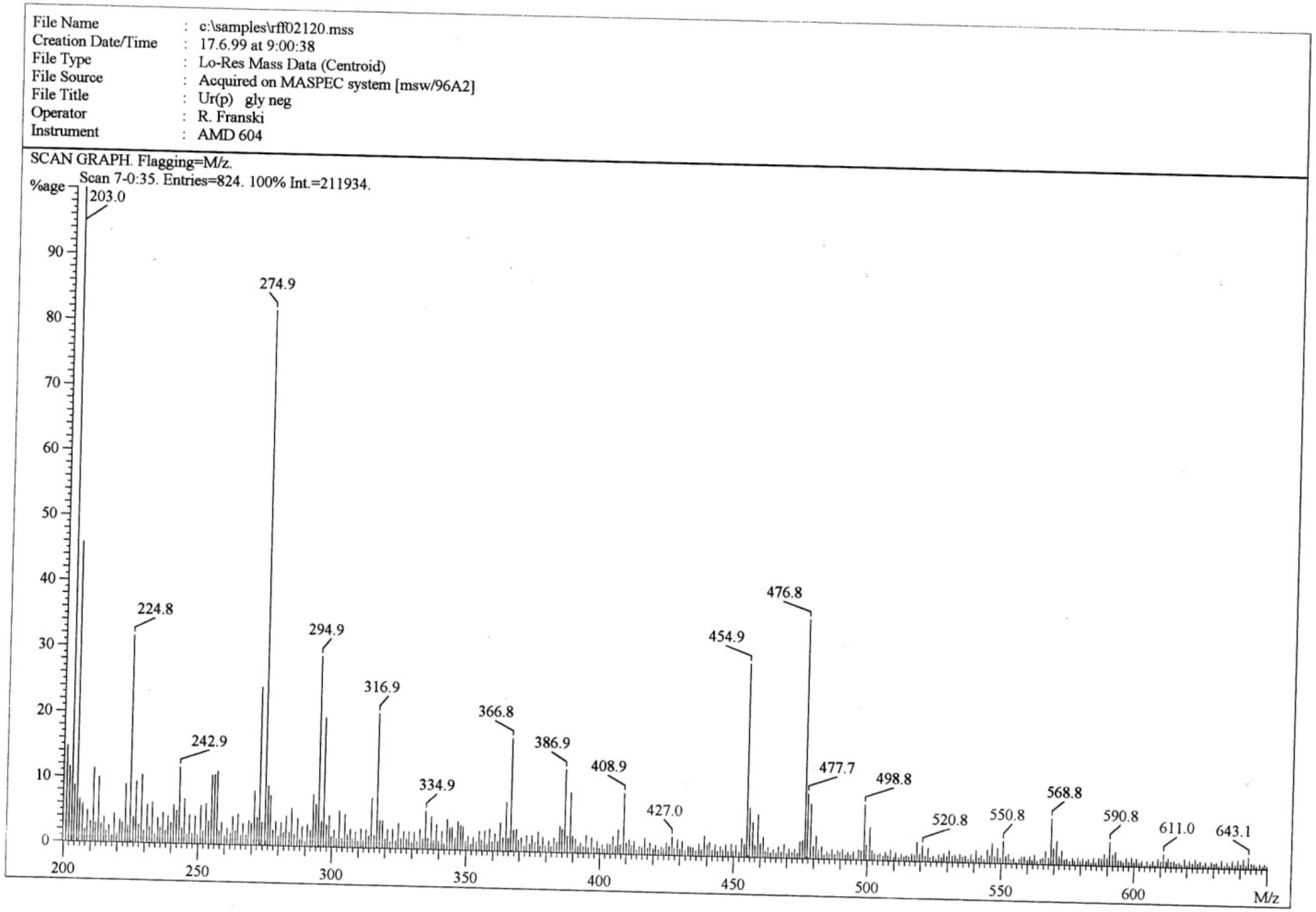

Figure S22. Atomic composition of Compound **7d.**

ASD01828.TXT

ATOMIC COMPOSITION REPORT

```
File Name        : c:\samples\test1.mss.
File Date/Time   : 17.6.99 at 9:10:22
File Type        : Hi-Res Mass Spectrum
File Source      : Acquired on MASPEC system [msw/96A2]
                   as c:\samples\rfv02122.mss
File Title       : Ur (p)
Source File      : c:\samples\rfv02122.mss
Source Scan(s)   : 1:

Sort Field       : M/z (ascending).

Scan Filter      : none.
```

Selected isotopes:

Symbol	Min	Max	V'cy	Name
C	9	17	4	Carbon-12
H	10	22	1	Hydrogen-1
N	0	2	3	Nitrogen-14
O	0	13	2	Oxygen-16
Na	0	1	1	Sodium-23
P	0	1	3	Phosphorous-31

Allowable error = minimum of 10.0 ppm, 5.0 mmu.

Number of Peaks=36.
Base Peak=477.06033, 100% Int.=9866.

Mass	(%)	Calculated	ppm	mmu	Formula
455.07004	53.88	455.07031	0.6	0.3	C14.H20.N2.O13.P
477.05220	83.48	477.05225	0.1	0.0	C14.H19.N2.O13.Na.P

***** End of Atomic Composition Report *****

Figure S23. ^{31}P-NMR of Compound **9a.**

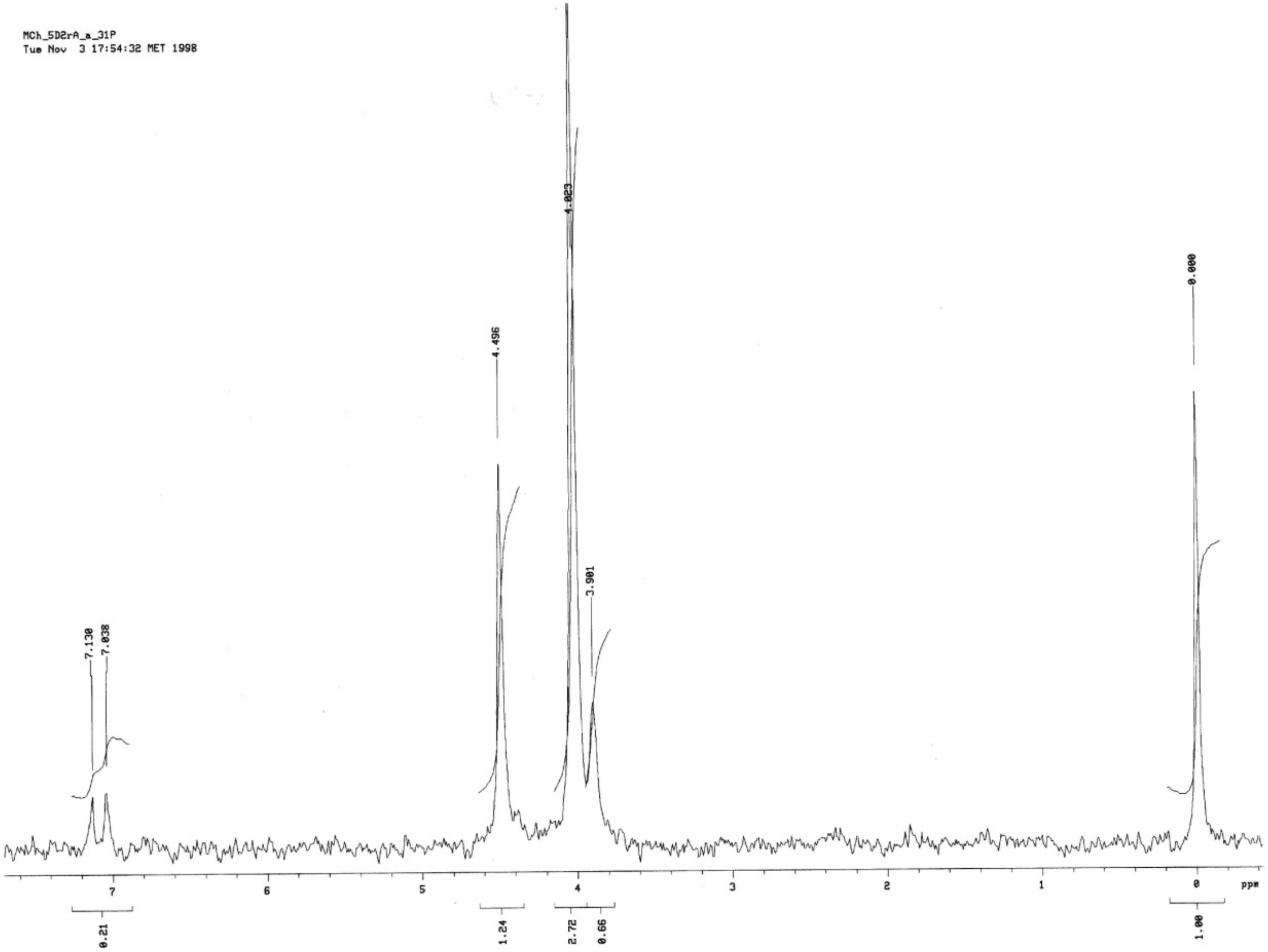

Figure S24. MS Analysis of Compound **9a.**

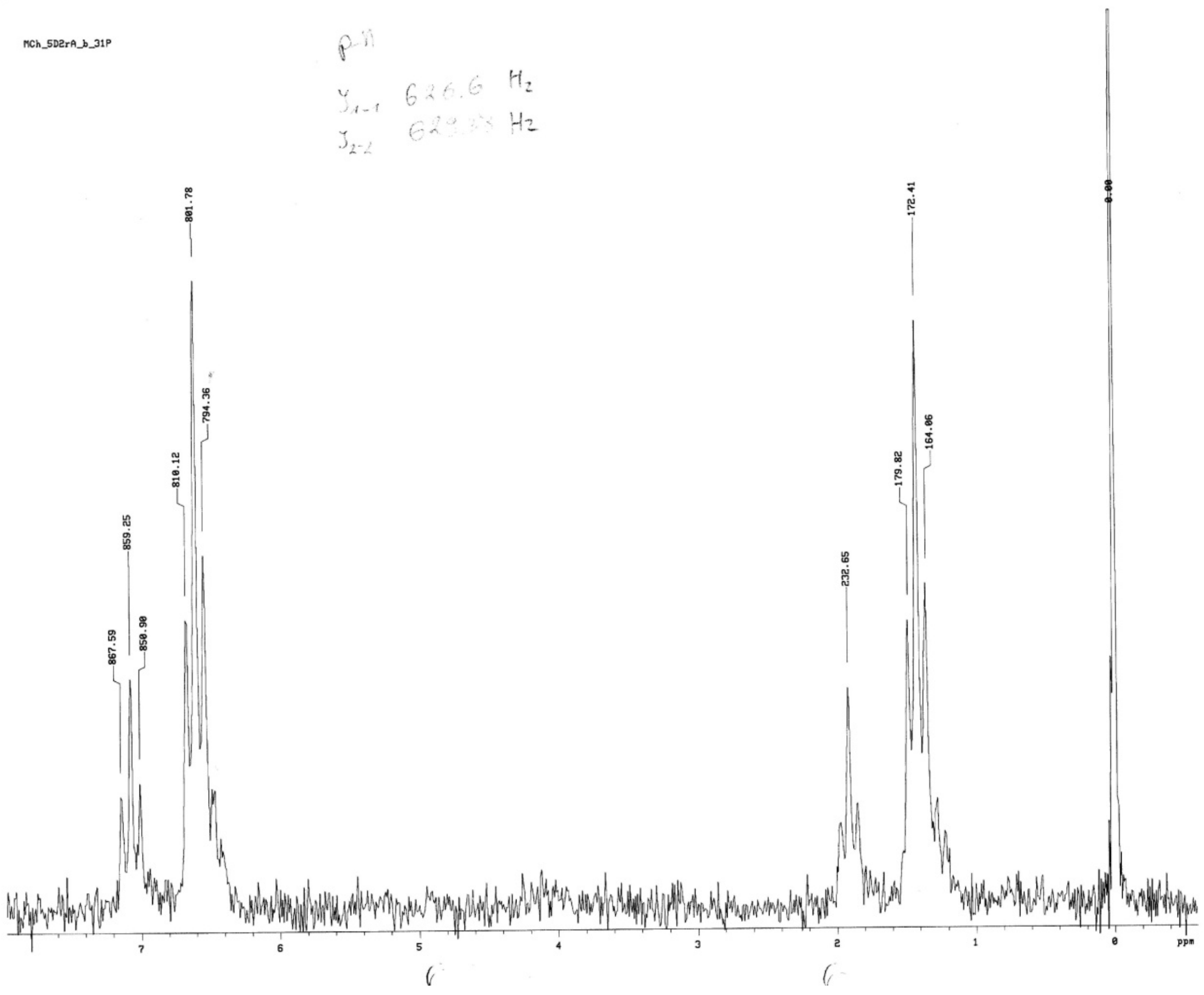

Figure S25. MS Analysis of Compound **9a.**

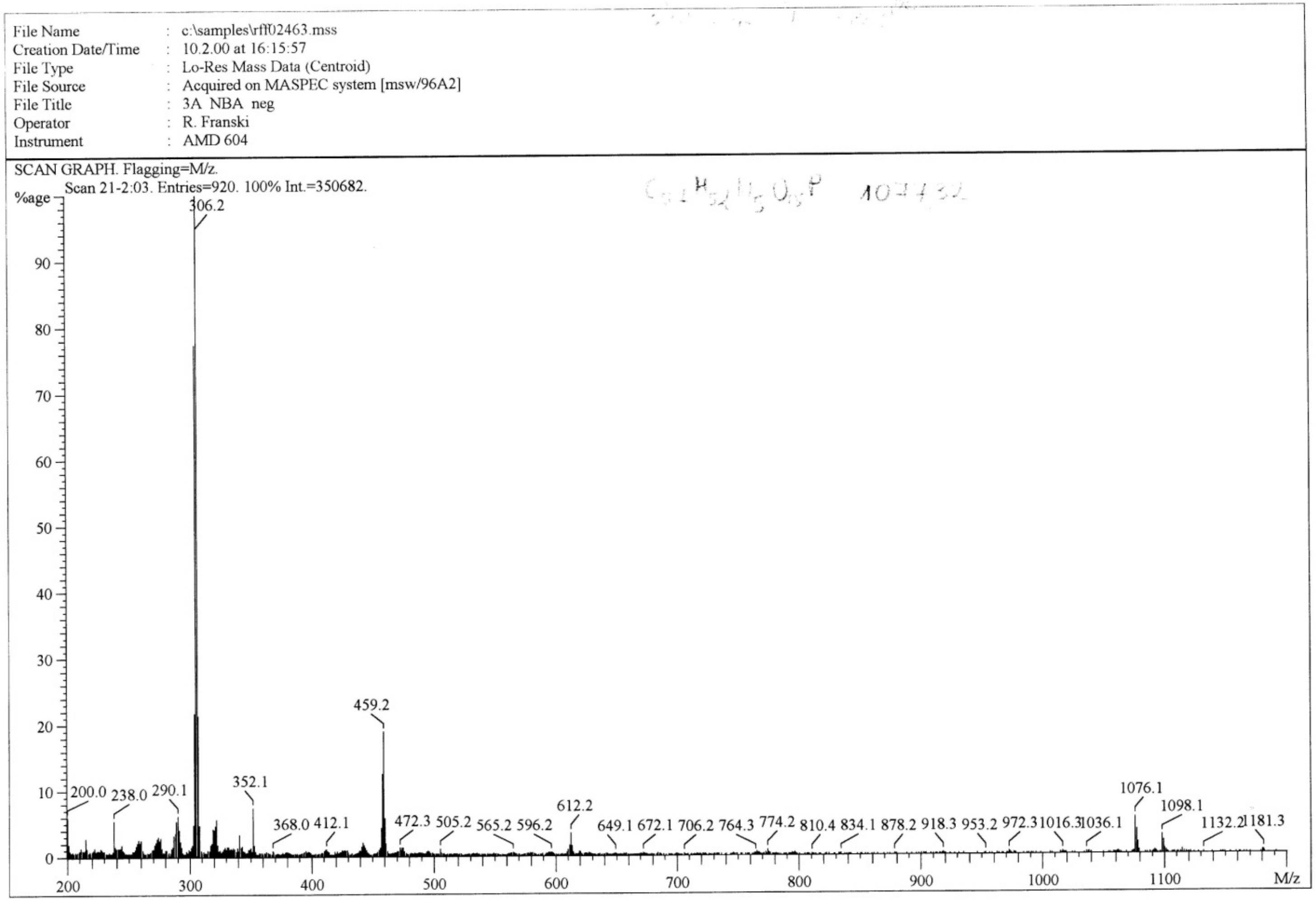

Figure S26. ^{31}P-NMR of Compound **9c**.

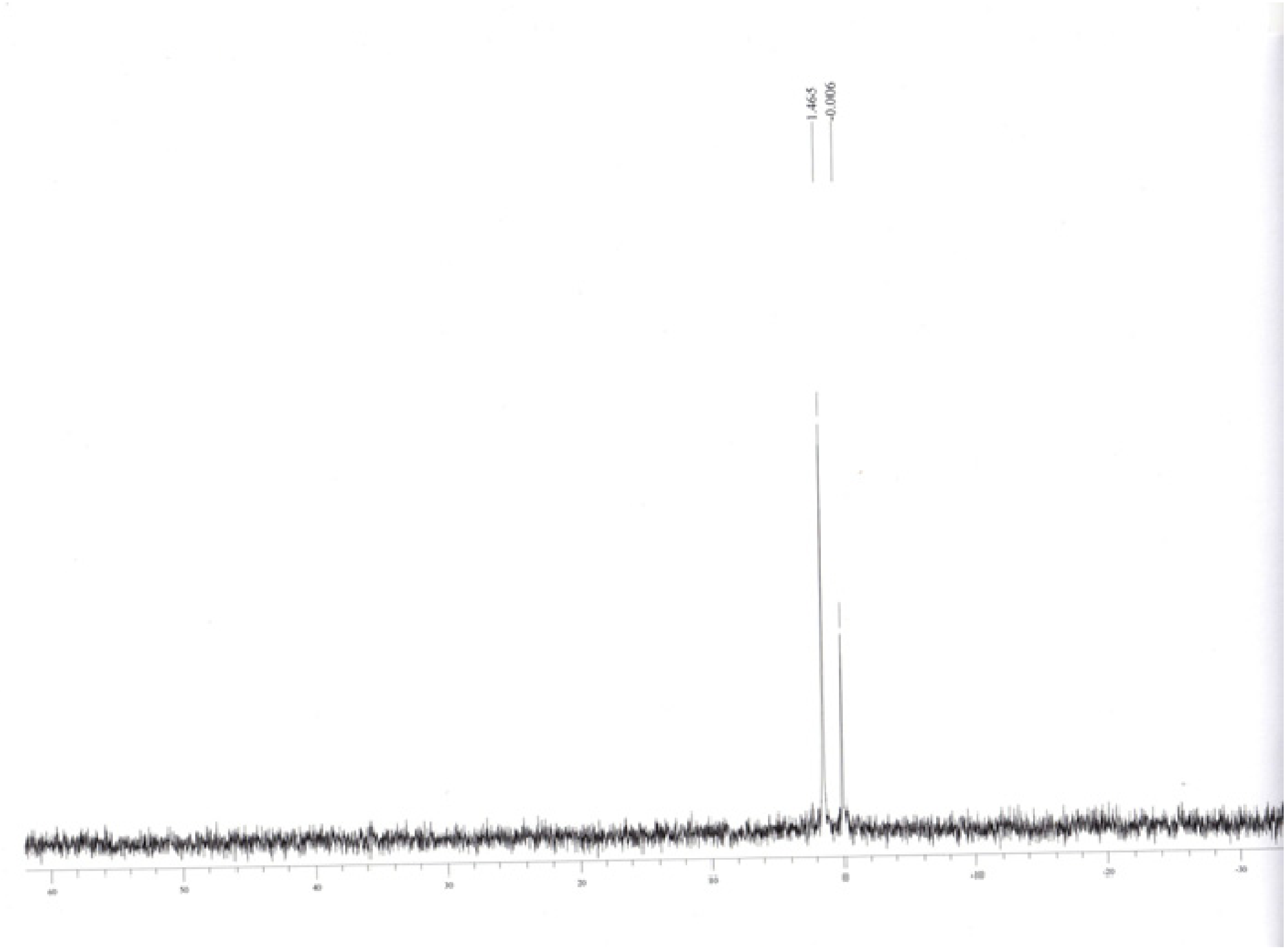

Figure S27. ^{31}P-NMR of Compound **9c**.

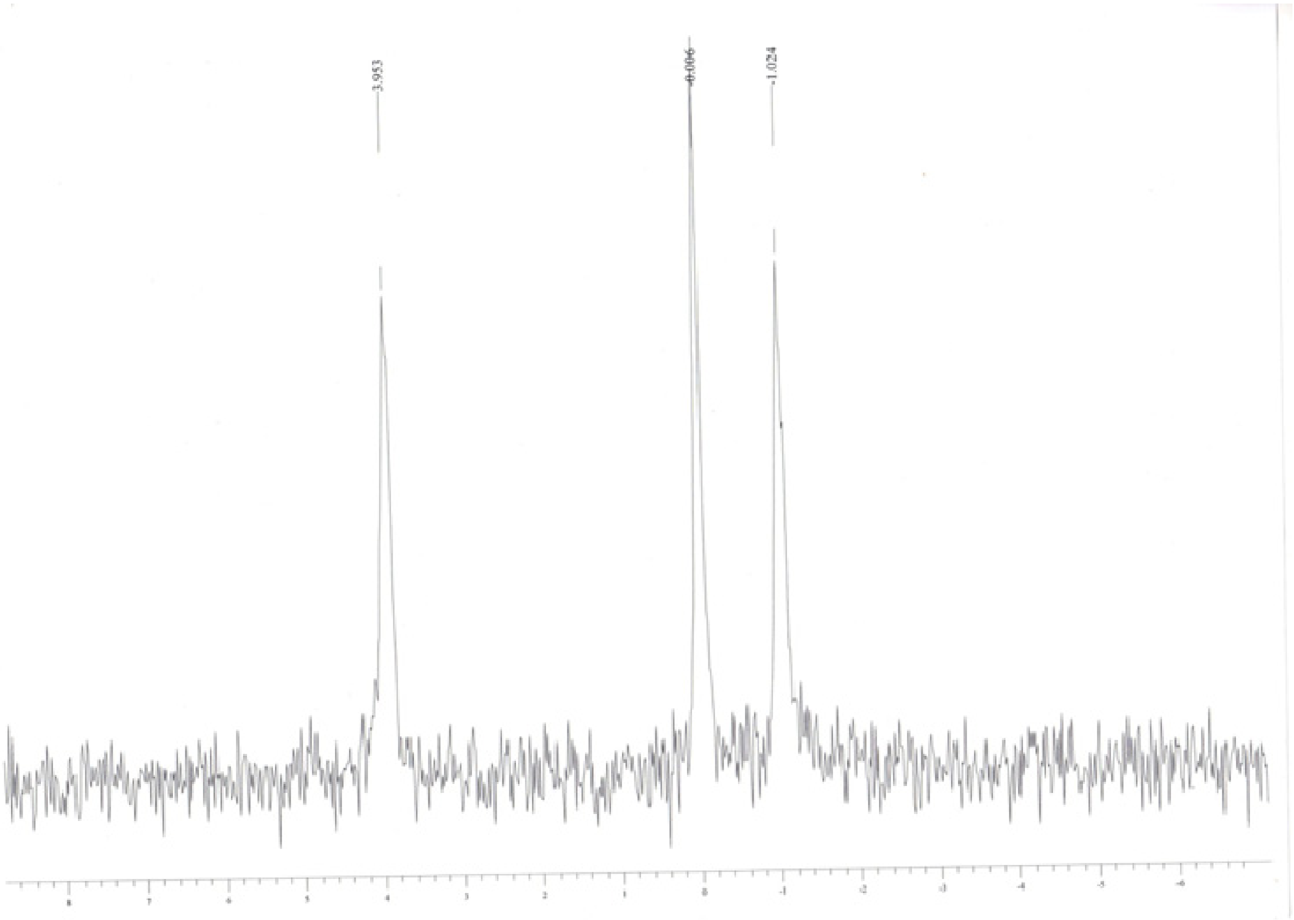

Figure S28. ^{31}P-NMR Compound **12a**.

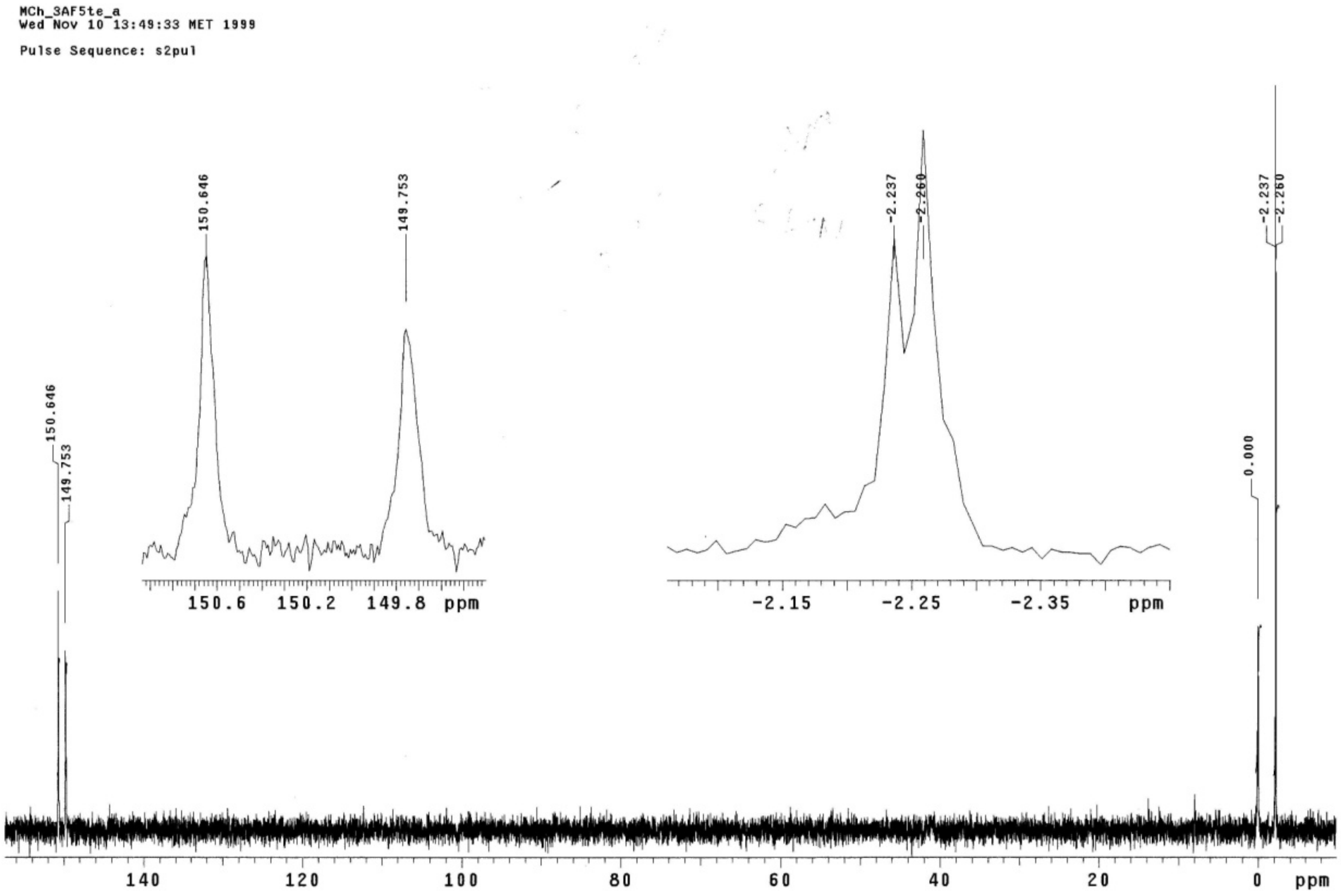

Figure S29. MS Analysis of Compound **12a**.

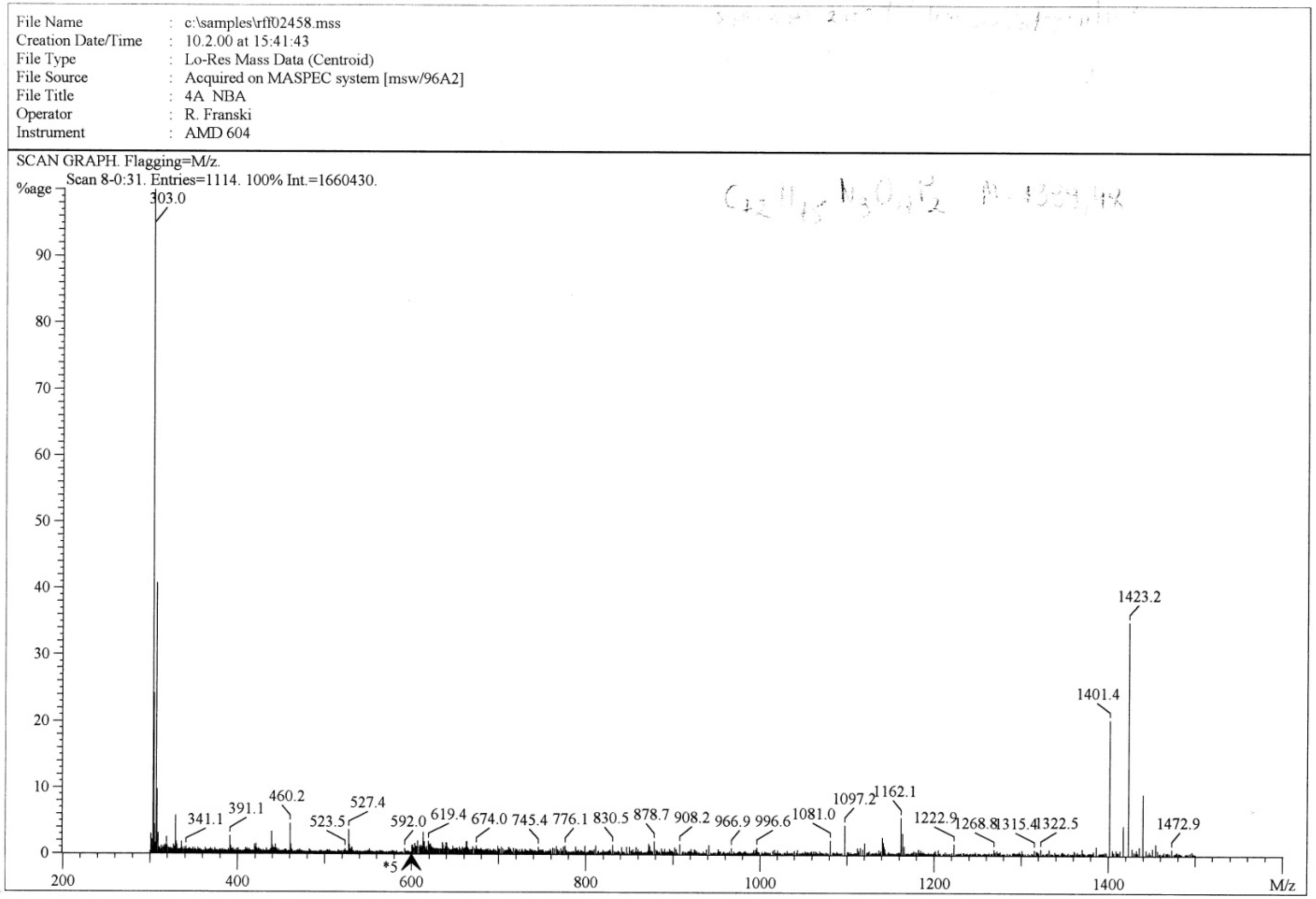

Figure S30. ^{31}P-NMR of Compound **12c**.

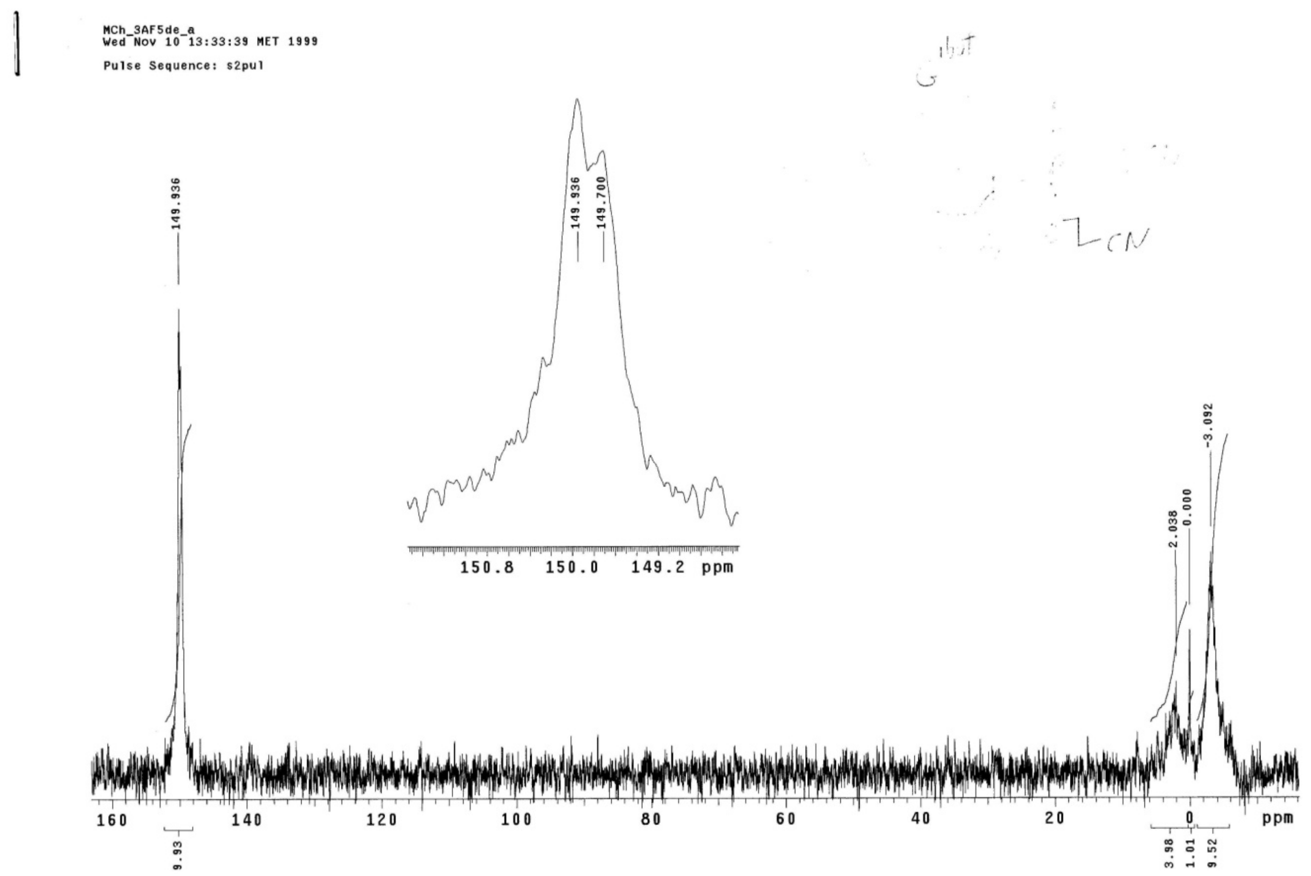

Figure S31. MS Analysis of D1.

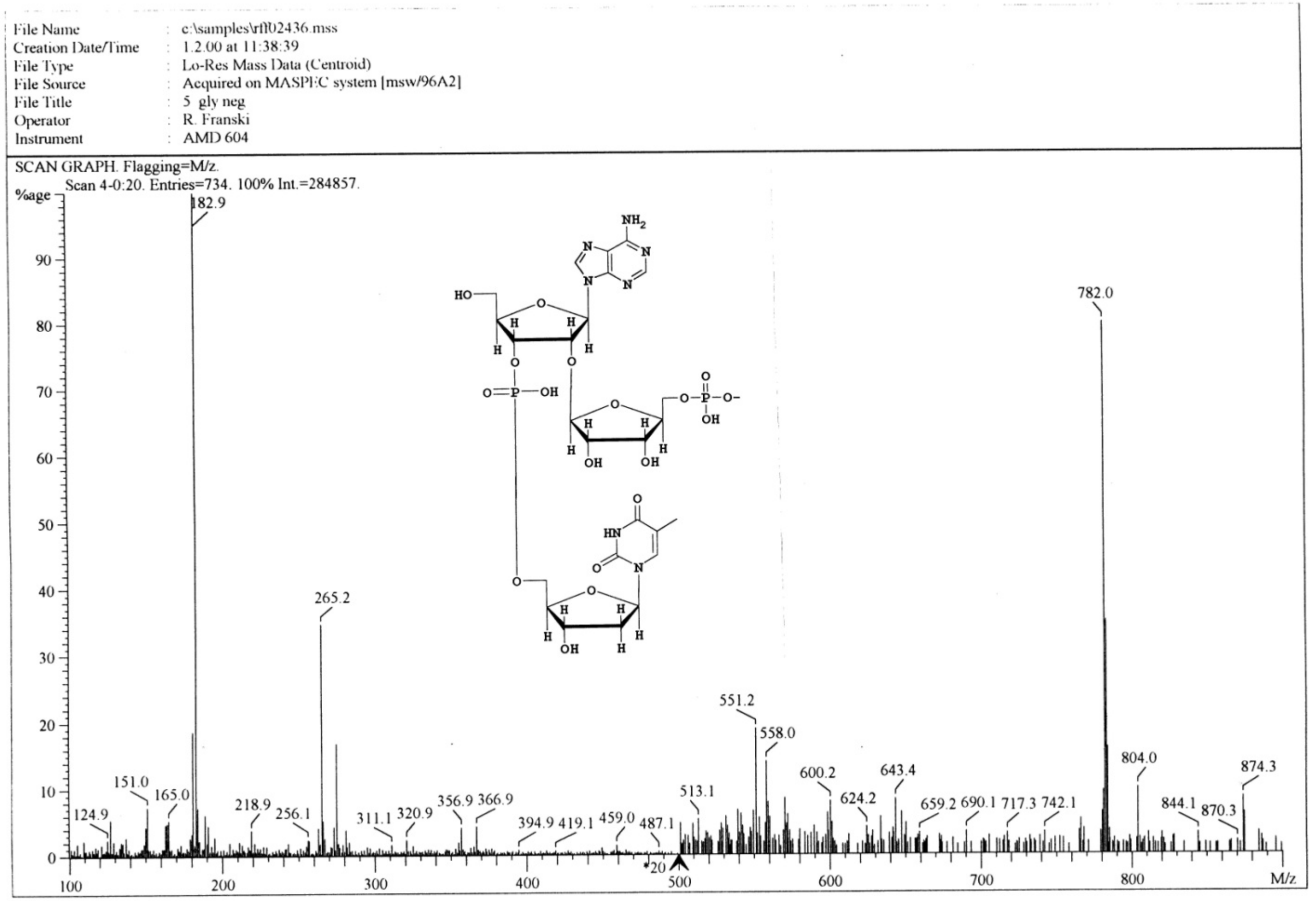

Figure S32. MS Analysis of D2.

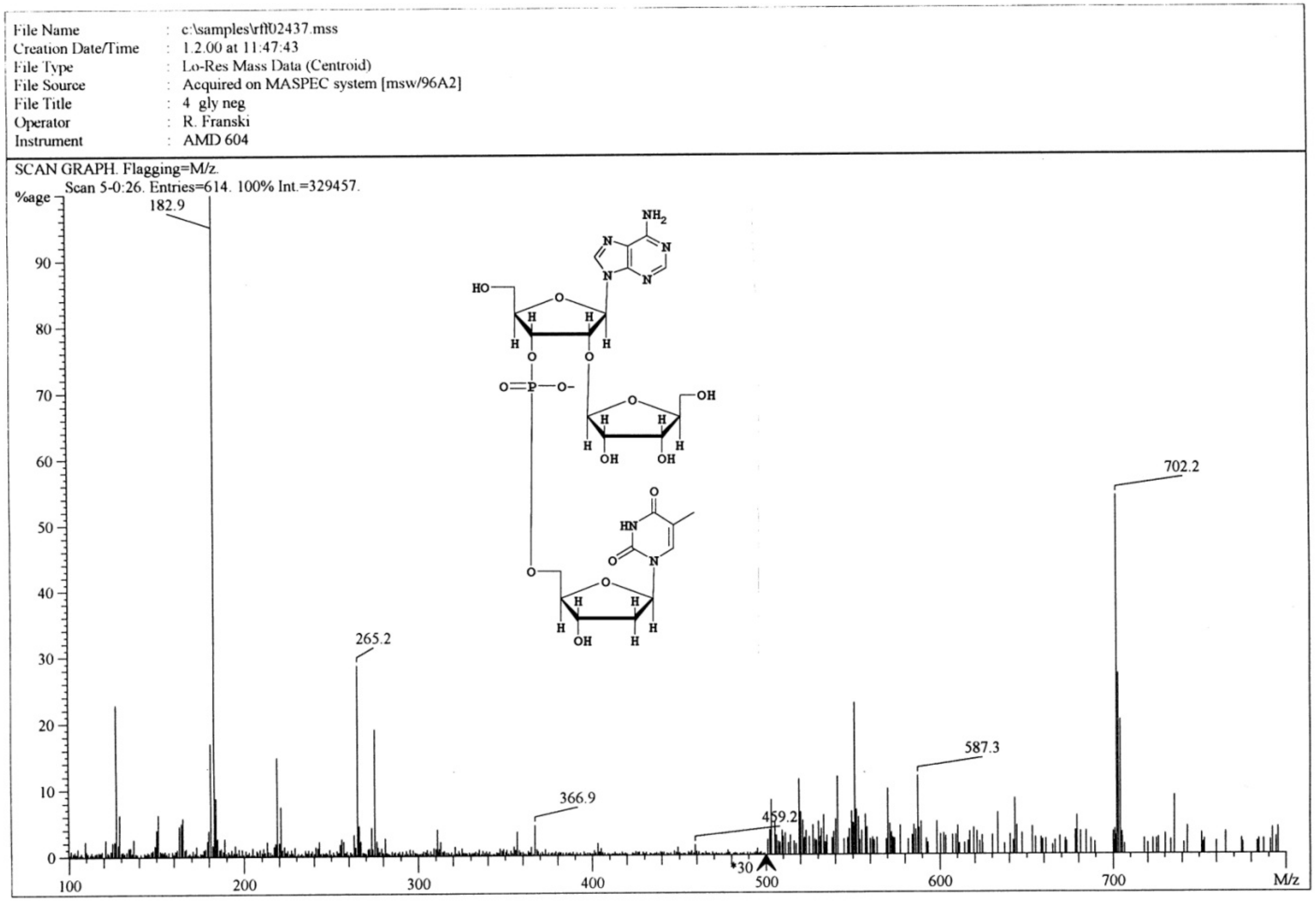

Figure S33. Determine the purity of dimer D1 and D2 by HPLC analysis

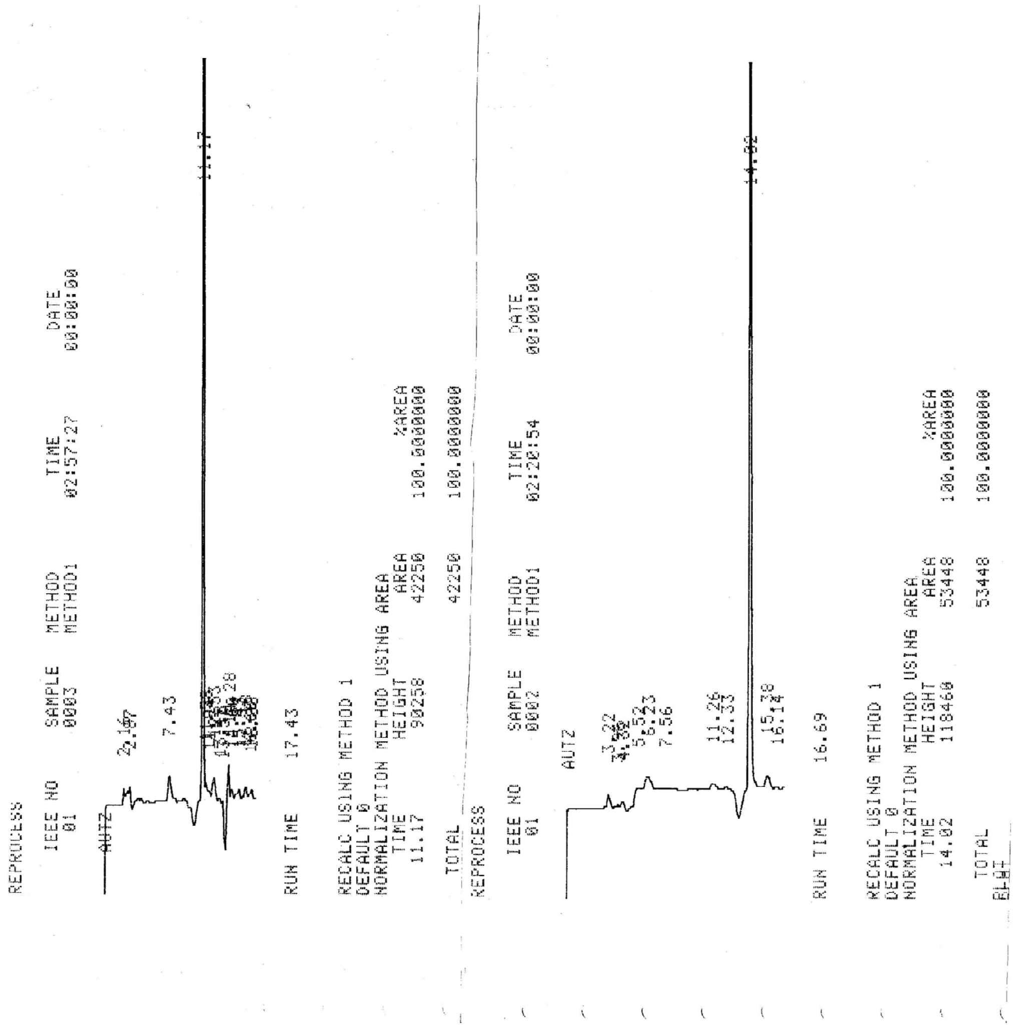

Supplement: Supplementary file 1 [file molecules-18-14780-s001.pdf]
